# A novel machine learning approach reveals latent vascular phenotypes predictive of renal cancer outcome

**DOI:** 10.1038/s41598-017-13196-4

**Published:** 2017-10-16

**Authors:** Nathan Ing, Fangjin Huang, Andrew Conley, Sungyong You, Zhaoxuan Ma, Sergey Klimov, Chisato Ohe, Xiaopu Yuan, Mahul B. Amin, Robert Figlin, Arkadiusz Gertych, Beatrice S. Knudsen

**Affiliations:** 1 Department of Surgery, Cedars Sinai Medical Center, Los Angeles, CA USA; 2 Department of Biomedical Sciences, Cedars Sinai Medical Center, Los Angeles, CA USA; 3 Department of Pathology, Cedars Sinai Medical Center, Los Angeles, CA USA; 4 Samuel Oschin Comprehensive Cancer Institute, Cedars Sinai Medical Center, Los Angeles, CA USA

## Abstract

Gene expression signatures are commonly used as predictive biomarkers, but do not capture structural features within the tissue architecture. Here we apply a 2-step machine learning framework for quantitative imaging of tumor vasculature to derive a spatially informed, prognostic gene signature. The trained algorithms classify endothelial cells and generate a vascular area mask (VAM) in H&E micrographs of clear cell renal cell carcinoma (ccRCC) cases from The Cancer Genome Atlas (TCGA). Quantification of VAMs led to the discovery of 9 vascular features (9VF) that predicted disease-free-survival in a discovery cohort (n = 64, HR = 2.3). Correlation analysis and information gain identified a 14 gene expression signature related to the 9VF’s. Two generalized linear models with elastic net regularization (14VF and 14GT), based on the 14 genes, separated independent cohorts of up to 301 cases into good and poor disease-free survival groups (14VF HR = 2.4, 14GT HR = 3.33). For the first time, we successfully applied digital image analysis and targeted machine learning to develop prognostic, morphology-based, gene expression signatures from the vascular architecture. This novel morphogenomic approach has the potential to improve previous methods for biomarker development.

## Introduction

Analytical strategies involving machine learning have recently been applied to biomarker discovery in digital pathology images. One machine learning approach, broadly called Deep Learning, involves automatic recognition of cancerous tissue with millions of discrete parameters, hindering reasonable interpretation of discriminative features. In another branch of machine learning, algorithms are developed to recognize specific predefined features in images and measure their abundance. The latter approach permits algorithm design informed by observations related to biological concepts, making it the preferred strategy for analyzing multicellular biological processes^1–15^.

Important prognostic information has been obtained from analysis of RNA expression data through measurements of pathways that drive cell intrinsic biological mechanisms such as transcription factor activity, stemness, epithelial-to-mesenchymal transition or neuronal differentiation^16^. Unfortunately, this approach is confounded by the averaging of signals across heterogeneous cell types and across the ternary spatial organization of higher order structures from which the RNA is obtained. These spatial relationships are indispensable to diagnostic interpretation by pathologists but are difficult to quantify without computational assistance.

Recent computational and machine learning tools provide new opportunities to quantify the cellular composition and spatial organization of the tumor and its microenvironment (TME)^2,13^. Despite the importance of angiogenesis in the TME for tumor growth and aggressiveness, the tumor vasculature has been incompletely represented by both image analysis and gene expression analysis. Since the tumor vasculature is a highly orchestrated network of branched tubular structures, it is useful as a model system to determine how higher order cellular structures may be captured through linking quantitative imaging with genomic data.

In clear cell Renal Cell Carcinoma (ccRCC), the most common subtype of renal cell carcinomas^17^, excessive angiogenesis constitutes a pathognomonic diagnostic feature. It is caused by the loss of the Von Hippel Lindau tumor suppressor protein, VHL, which results in secretion of vascular endothelial growth factor (VEGF)^18^. Anti-vascular agents have been approved by the FDA for treatment of ccRCC^19^, attesting to the key role of the vasculature in tumor growth and metastatic progression^20–22^. Tumor angiogenesis involves complex and multicellular interactions that include intercellular signaling between budding tip cells, proliferating stalk cells and supporting perivascular cells^23^. Since multiple cell types cannot be distinguished through genomic analyses, vascular gene expression signatures fail to capture the dynamics of tumor angiogenesis that ultimately defines the configuration of the vascular network^24^. While existing vascular signatures can be used to determine the magnitude of tumor vascularization, they are not able to detect differences in the vascular structures amongst cancers.

We hypothesized that a machine learning approach could be used to capture prognostic information embedded in the spatial organization of the vascular networks within ccRCC. Furthermore, we hypothesized that gene expression signatures carrying nascent spatial information would lead to prognostic information for patients. To test these hypotheses, we pursued an integrated digital image analysis and computational biology approach in which we derived a gene expression signature from phenotypic vascular features.

## Results

The vasculature of renal tumors consists of a complex array of branched vascular channels that are generated in response to the secretion of vascular endothelial growth factor by cancer cells. They are surrounded by extracellular matrix and perivascular cells to form structures that vary in thickness, length and cell density. The formation of the vascular network is orchestrated through interactions between vascular cells and cancer cells. The complexity of these interactions is not understood at the gene expression level, but is reflected in the vascular architecture. Therefore vascular heterogeneity is attributable to tumor specific gene expression.

**Figure 1:**
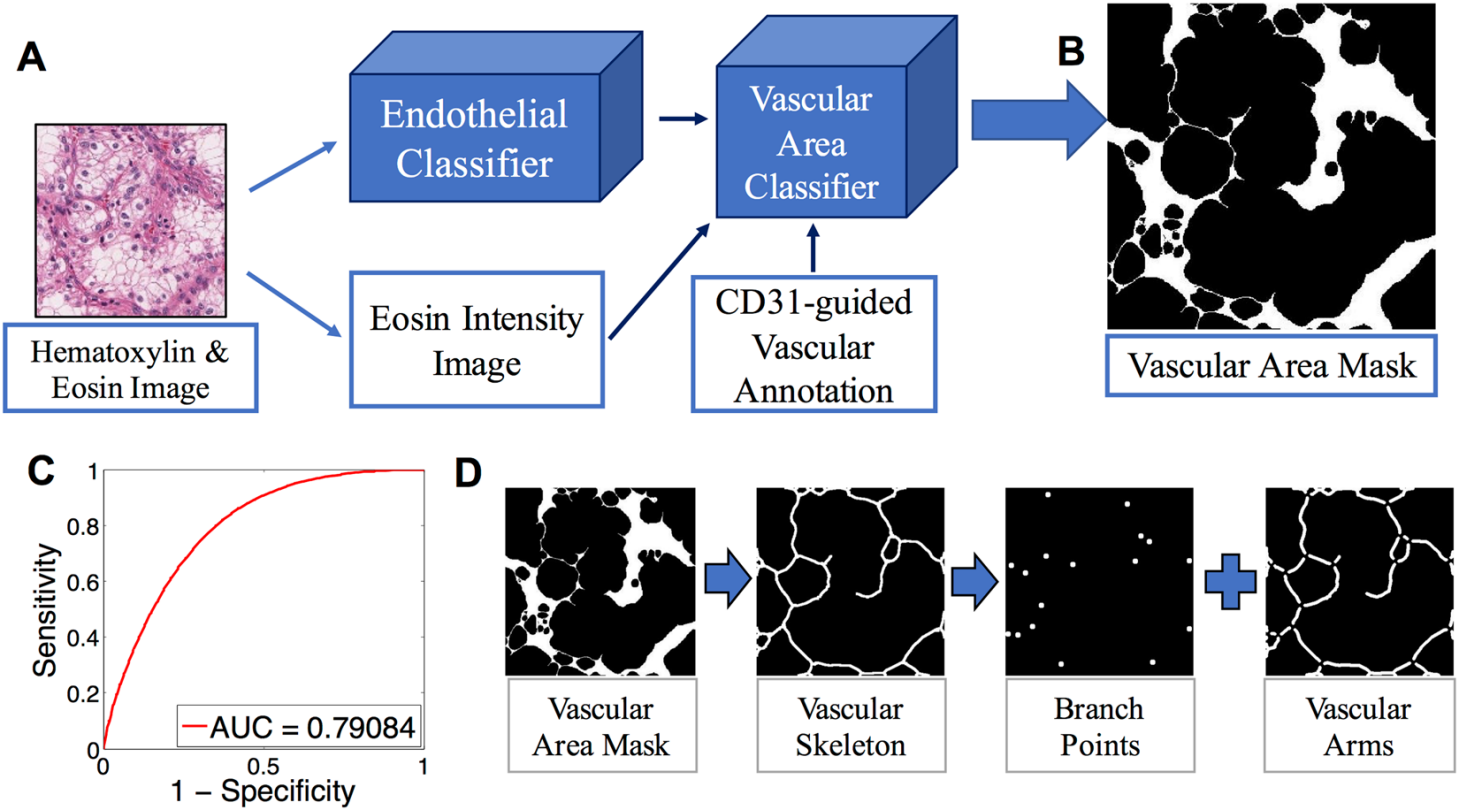
Vascular area delineation in H&E stained slides using sequential, machine learning-based, 2-step vascular area classification approach. (**A**) Training of the first classification model: The original H&E image is processed into an endothelial nuclear mask based on hematoxylin staining and an eosin intensity image. The digital image of CD31 immunohistochemistry provides vascular annotation to train the vascular area classifier. (**B**) The second classifier outputs a vascular area mask (VAM) where vascular areas are white, and non-vascular areas are black. (**C**) Receiver Operating Characteristic (ROC) curve for vascular area classification compared to the vascular annotation provided by CD31 immunohistochemistry (AUC = 0.78). (**D**) Post-processing of the VAM.

To analyze the vascular network of ccRCC, we developed an automated method for outlining the vasculature in digital images of H&E slides (Fig. 1). Two machine learning tools were developed, the first tool to locate endothelial cells (EC), and the second tool to delineate blood vessels and perivascular areas. To automate the annotation of EC nuclei and improve machine learning accuracy, we transferred cell type information from slides stained by immunohistochemistry (IHC) to H&E stained images of the exact same tissue section (Fig. S1). After generating a digital image of the H&E slide, the tissue was decolorized and individual EC or lymphocyte nuclei were marked by CD31 (purple) and CD45 (brown), respectively (Figs S2, S3). The CD31 mask was automatically generated with a custom image analysis software (Data File 1) and transferred to the H&E image by co-registration of IHC and H&E images. By this method ECs and lymphocytes in H&E stained digital slides of 8 cases of ccRCC were identified and annotated based on the reactivity of cells with CD31 and CD45 antibodies.

### Machine learning enables vascular morphometry in H&E stained slides

We trained a first SVM classifier on 63 morphometric and texture features (Fig. S1; Table S1)^8,10,15^, to specifically detect endothelial cells (EC) in H&E images based on the annotation of 22,600 nuclei (6,000 endothelial, 600 lymphocyte, 16,000 cancer; Table S2). The classifier performed well to separate EC, lymphocyte, and cancer nuclei in a testing set of different H&E images from the same cases, which contained over 270,000 nuclei (77,500 EC, 9,000 lymphocyte, 183,500 cancer; Cohen’s Kappa = 0.68, Observed agreement = 0.87). Altogether, this standard supervised classification method trained through IHC annotation accurately classifies EC nuclei (AUC for EC prediction = 0.95) and masks of classified EC nuclei were used to seed the vascular network analysis.

Next, we trained a second classifier that delineates vascular areas composed of EC and perivascular cells embedded in a collagenous, eosinophilic extracellular matrix (Fig. 1A). For training, we combined the binary mask of classified EC nuclei, the eosin intensity image and the CD31 vessel mask. Each pixel in the unmixed^25^, normalized and smoothened eosin image was expressed as a vector of 17 features that included information of nearby EC locations, and the intensity of eosin in its immediate area^26^ (Figs S5, S6). Vectors were labeled as originating from either vascular or non-vascular pixels by using the CD31 vessel mask as a reference. We observed an overall difference in the vectors from pixels in CD31 positive areas compared to those from CD31 negative areas (n = 6,000 Wilcoxon Rank-Sum test *p* < 0.001; Fig. S7). A Random Forest classifier was used to identify vascular and non-vascular areas based on 6,000 randomly sampled pixels (Supplementary methods). This pixel-wise classification ultimately results in a binary (black and white) image of tumor vasculature that we named the vascular area mask (VAM) (Fig. 1B). The accuracy of the VAM was assessed by referencing annotated IHC images in a testing set (AUC = 0.79; Fig. 1C).

In addition to the binary VAM dataset, we analyzed datasets of EC nuclei, branch points of vascular arms (BP) and the vascular arm structures without perivascular cells (Fig. 1D). In each of the 4 data sets, 22 primary image features were extracted including the white object area, object density, solidity, eccentricity, Euler-Poincare characteristic^27^, fractal dimension^28^, and lacunarity (Fig. S8). The distribution of each feature in the image tile was captured by its mean, standard deviation, skewness and kurtosis, jointly resulting in 88 vascular features (VF).

### Vascular features predict disease free survival

**Figure 2:**
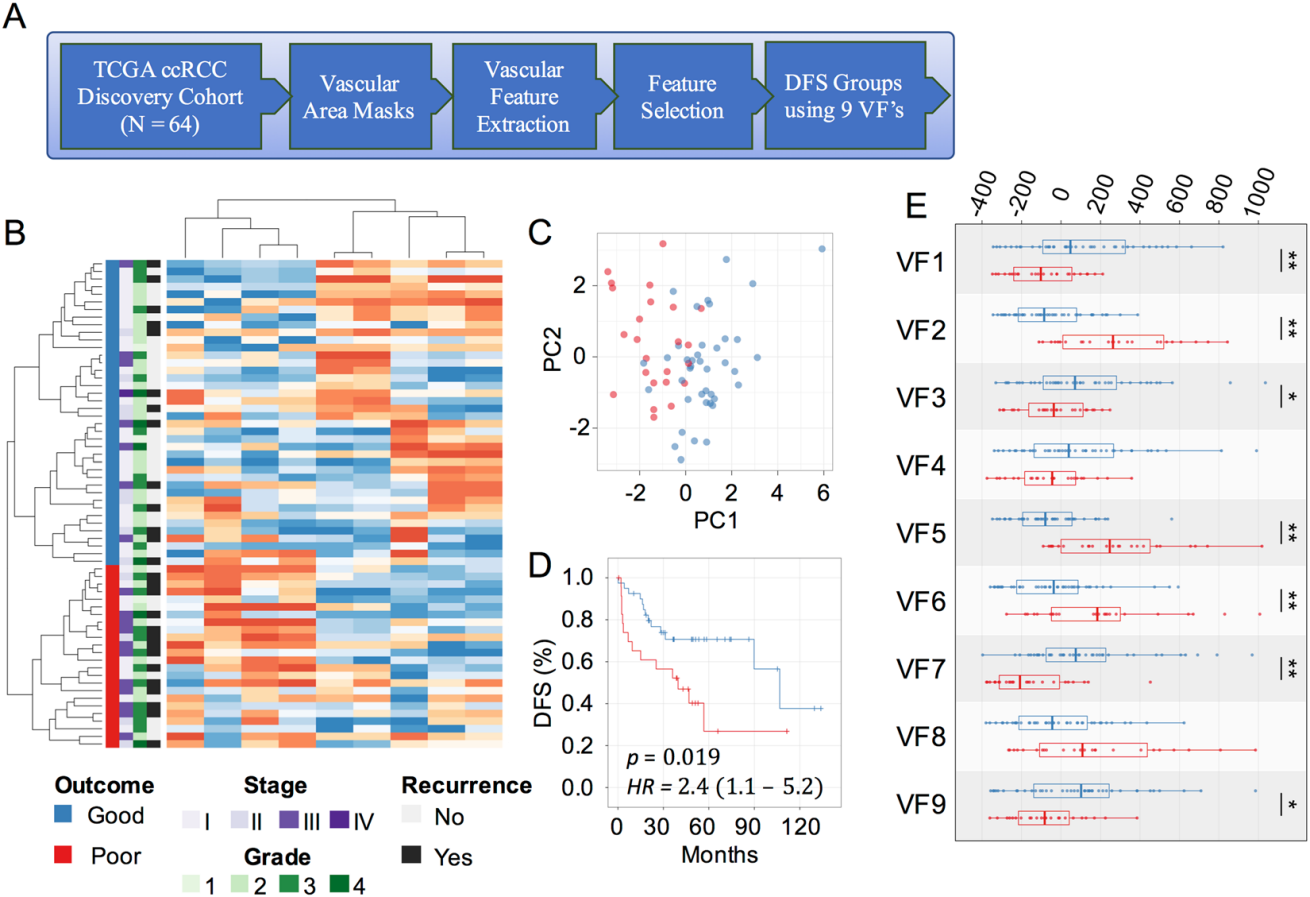
9 vascular features (VF) predict disease free survival (DFS) in ccRCC. **(A)** Workflow diagram for predicting outcomes with selected vascular features in the ccRCC discovery cohort (N = 64). **(B)** Hierarchical clustering of the 64 cases by 9 vascular features into two risk groups. Blue – low risk, red – high risk. **(C)** Principal component analysis demonstrating the first two principal components of the 9 selected VF’s. **(D)** Kaplan-Meier plot using 9VF’s to separate patients into low- and high-risk groups (*HR* = 2.4, *95% C.I*. = 1.1–5.2). **(E)** Box plot of VF expression in good and poor DFS risk groups (two-tailed t-test *p < 0.1, **p < 0.05). The 9VF’s are described in Table 1 and Supplementary Table S7.

Our ultimate goal was to identify a prognostic gene expression signature that is associated with vascular morphology. This required using a cohort of high quality images, RNA expression data, and patient outcome information. Based on these selection criteria we identified a discovery cohort of 64 cases within the Cancer Genome Atlas (TCGA)^17^ (Fig. 2A). To avoid artifacts, the tumor area was outlined by hand and between 3 and 74 (mean = 25, std = 18) image tiles per case were obtained, encompassing the entire tumor area that was suitable for analysis. For each tile, we calculated a primary feature value for each of 22 features. For each feature, the distribution across all tiles from a case was described through 4 secondary features. Thus, altogether 88 VFs were extracted from the tumor area of each case. Elimination of VFs with low variance (std/mean < 0.3) provided a set of 56 features for analysis. Next, we identified a subset of 9 VFs that optimally stratified discovery cohort cases into high risk and low risk groups. These VFs were selected through a sequential backwards feature selection that aimed to minimize the log-rank *p*-value between the survival curves of the two groups^29^. Unsupervised hierarchical clustering of cases using the 9 VFs divided the cohort into two clusters (Fig. 2B,C) and the number of clusters was confirmed by consensus clustering (Fig. S9). Cases within these clusters had a significantly different DFS probability by Kaplan-Meier analysis (log-rank *p* = 0.019, HR = 2.4, 95% C.I. = 1.1–5.2; Fig. 2D). The 9 VFs included 4 secondary features of EC spatial density and texture, 4 secondary features of vascular arm texture, and 1 feature of BP arrangement. To assess the effect of intra-tumoral heterogeneity on these features we performed an F-test that revealed the intra-case variance was significantly less than the inter-case variance (p < 0.001) for all the selected features (Fig. S10). Their mean expression values differed significantly between low risk (n = 40) and high risk (n = 24) groups (two-tailed t-test *p* < 0.05). Four features (EC lacunarity, arm orientations, BP lacunarity, and EC density) had greater standard deviations in the poor relative to good outcomes cases (Figs 2E, S11). The intra-tumor variance of these features is related to the heterogeneity in vascular organization and spatial aggregation of endothelial cells and vascular structures, consistent with the concept that disordered vascular architecture is associated with adverse outcome (Tables 1, S7). In contrast, the good prognosis cases were characterized by greater consistency of vascular organization across tiles, which led to more outliers in the distribution of 3 features (EC density, arm number and arm lacunarity). Thus favorable prognosis maybe related to greater overall vascular density and a larger number of tiles containing hot and cold spots of vascular density. Since the 9 VFs are associated with the heterogeneity of the vascular architecture within individual tiles, the tile size may affect the value of VFs in the 9 VF signature.

**Table 1:** Associations between Vascular Features (VFs) and genes in the 14GT signature. The 9 VFs and correlated genes are divided based on their higher average expression in good versus poor outcomes groups. While VFs associated with poor prognosis demonstrate high intratumoral variance (standard deviation), those associated with good prognosis indicate a relationship between high vascularity and hot/cold spots of vascular density and favorable prognosis.

	Standard Deviation of Features	Correlated genes
**Associated with Poor Outcome**	Arm orientations*	ADH5, NLRC4, RPL36A, RPLP2, SLC16A4, TNFSF8, ZNF16, SGCB, GOSR2
	BP Lacunarity*	
	EC Lacunarity	
	EC Density*	
	**Skewness (S) and Kurtosis (K) of feature distribution**	**Correlated genes**
**Associated with Good Outcome**	Arm number (S*, K)	IFNA13, CMYA5, STAT3, KCNJ12, MED10
	EC Density (S, K*)	
	Arm Lacunarity (K*)	

Asterisks (*) indicate VFs that correlate with genes in the 14GT signature.

### A gene expression profile derived from vascular features predicts survival

**Figure 3:**
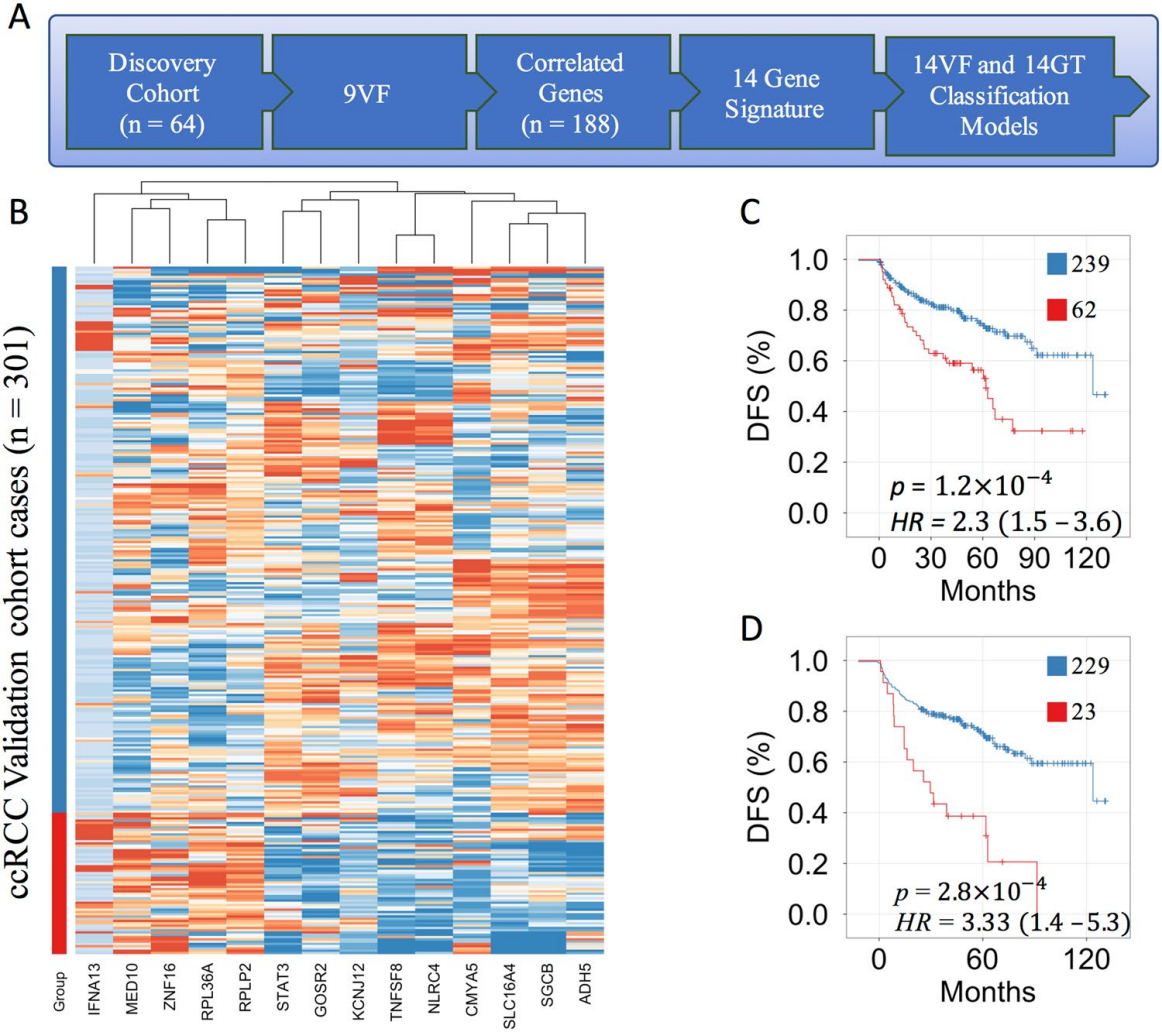
A 14-gene expression signature predicts disease free survival in ccRCC. **(A)** Workflow diagram of selecting 14 genes correlated to 9 VF’s and training of two outcomes prediction models in the discovery cohort (N = 64). The 14VF classifier was trained on the risk groups defined by 9VF’s. The 14GT classifier was trained on 24-months disease free survival status. **(B)** Supervised clustering based on low risk (blue) and high risk (red) groups obtained from the 14VF model in the validation cohort (N = 301). **(C)** Kaplan-Meier plot of the 14VF model (n = 207, log-rank test *p* < 0.001, *HR* = 2.3). **(D)** Kaplan-Meier plot of the 14GT model (n = 257, log-rank test *p* < 0.001, *HR* = 3.33).

Prognostic gene expression signatures are typically identified by comparing gene expression profiles between good and poor prognosis groups. Since gene expression is measured in dissociated tissues, information about the tissue architecture is lost. To obtain a gene expression signature that contains the spatial information of the vascular network, we first identified a set of genes correlative to the 9VF’s (Fig. 3). Within the discovery cohort, all genes (n ~ 20,000) were ranked based on Pearson’s correlation coefficient with each VF. The top 0.05% positively and negatively correlated genes were selected for each of the 9 VF’s, providing a set of 182 unique candidate genes (Table S4). To select a final gene signature we used the information gain ratio^30^ of each candidate gene with respect to VF-risk group assignment. Amongst the 182 genes, only 14 genes possessed a positive information gain and were included for further analysis (Tables 2, S5). The 14 genes correlated with 6 of the 9 VF’s. Eleven of 14 genes were significantly differentially expressed between high and low risk groups (Wilcoxon rank-sum test *p* < 0.05; Fig. S13). A heat map was used to visualize differences in expression levels of the 14 genes between the high and low VF-risk groups (Fig. S12). Differences in gene expression were also apparent in a multidimensional scaling plot and box plots (Fig. S13). The expression of 8 genes correlated with the vascular features representing vascular arm arrangement (“standard deviation of arm orientations”), 3 genes correlated with VFs describing the EC distribution and 3 genes correlated with vascular organization (lacunarity). Interestingly, 8 genes were functionally associated with vascular or cardiac development and 1 of the genes was previously demonstrated to predict the outcome of ccRCC^31^ (Table 2).

**Table 2:** Functional annotation of genes in 14 genes correlated to 9VFs and recapitulating the risk prediction of the 9VFs. Annotation of genes was performed manually through PubMed. Associated citations may be found as part of Supplementary Table S5.

	Gene name	Activity	Disease association
CMYA5**	Cardiomyopathy-associated 5	Desmin binding, Vesicular transport	Cardiomyopathy, schizophrenia
STAT3**	Signal transducer and activator of transcription 3	Signal transduction, Gene transcription	Angiogenesis, Vascular leakage
ADH5**	alcohol dehydrogenase 5	Opposes NO signaling, protein denitrosylation	Impaired cardiovascular function
NLRC4	NLR family CARD domain containing 4	Innate immunity inflammosome	Inflammatory disease, infantile enterocolitis
RPL36A***	Ribosomal protein L36	60S ribosomal subunit Translational regulation	Hepatocellular carcinoma
RPLP2***	ribosomal protein lateral stalk subunit P2	Phosphoprotein involved in protein elongation	Upregulated in many cancers
SLC16A4*	Solute carrier family 16 member 4	Monocarboxylate transporter for pH and energy homeostasis	Prognostic biomarker in ccRCC
TNFSF8	tumor necrosis factor superfamily member 8	CD30 ligand	Inflammation
ZNF16	zinc finger protein 16	Transcription factor	Erythroid and megakaryocyte differentiation
IFNA13**	interferon alpha 13	Inflammatory/reproductive cytokine	Downregulated in dilated cardiomyopathy
SGCB**	Sarcoglycan beta	Dystrophin complex, sarcoglycan transport	Limb-girdle muscular dystrophy cardiomyopathy
KCNJ12**	potassium voltage-gated channel subfamily J member 12	Repolarization of cardiac muscle	Dilated cardiomyopathy
MED10**	mediator complex subunit 10	RNA Pol-II transcriptional regulation	Heart valve development
GOSR2**	golgi SNAP receptor complex member 2	Vesicular trafficking	Familial essential hypertension
*Prognostic renal cancer biomarker		**Association with vascular or heart biology	***Cancer association

Next we built two prediction models using the 14 genes. The first model was trained on the VF risk groups in the discovery cohort (Fig. 2). Comparing 6 classification methods^32^ with 10-fold cross validation, the overall best classification performance was achieved by a Generalized Linear Model with Elastic Net Regularization (GLMNET; Fig. S14). The 14-gene GLMNET classifier (14VF) was applied to a validation cohort (n = 301). All genes were differentially expressed (*p* < 0.01) between high and low VF-risk groups. A heat map, a multidimensional scaling plot, and principal component analysis illustrate the differences between the two groups (Fig. 3B; Figs S15, S16). The second prediction model using the 14 genes was trained on 59 cases in the discovery cohort, divided into high and low risk groups by 24-month disease free survival status. Cases with less than 24 months follow-up of DFS were excluded (n = 5). We trained a second GLMNET classifier (14GT) using 10-fold cross validation. The 14GT classifier was applied to the validation cohort of 252 cases after censoring at 24-months.

To compare the 14VF and 14GT prediction models we used Kaplan Meier plots. Both models resulted in a significant separation of low and high risk groups. The 14VF hazard ratio was 2.3 (*p* = 1.2e-4; Fig. 3C) and the 14GT hazard ratio was 3.33 (*p* = 2.8e-4; Fig. 3D). We used a multivariate Cox regression model that included clinical stage and Fuhrman nuclear grade to determine whether patient stratification based on both morphogenomic prediction models were independent of stage (Table S6). Both models improved the outcomes prediction based on stage alone (C-Index: Stage = 0.7, Stage + 14VF = 0.74, Stage + 14GT = 0.74). Adding the Fuhrman grade did not improve the outcomes prediction. In addition, both models performed similarly to a previously reported, non-overlapping, 34 gene panel (Clear Code 34)^33^ (C-Index: Stage + CC34 = 0.75). Collectively, these results demonstrate that spatial information from the vascular network provides a novel direction to biomarker development.

## Discussion

We have developed a prognostic gene expression signature based on the configuration of vascular networks in ccRCC. Analysis of gene expression data from tissue homogenates of ccRCC did not reveal prognostic information in vascular genes, perhaps because this approach does not capture the vascular architecture. To quantify the vascular structures in digital H&E images of ccRCC, two machine learning approaches were employed. Machine learning models were trained by transferring the nuclear outlines of ECs directly from IHC to H&E images. This information transfer, which can be multiplexed to include a variety of cell types, constitutes an inexpensive and efficient method for annotating cell types in the tumor microenvironment. The classification of ECs in ccRCC provided a starting point for generation of VAMs that were used to extract biomarkers of vascular morphometric features. The framework was deployed to extract vascular features from cases of ccRCC in the TCGA database. We identified 9 VFs that separated high risk and low risk groups in a discovery cohort (n = 64; HR = 2.4, 95% CI = 1.1–5.2). Two GLMNET classification models (14VF and 14GT), trained on 14 genes that were derived from correlation with the 9 VFs, were applied to cases in an independent TCGA validation cohort which did not possess adequate image quality to directly quantitate the vascular architecture. The gene classifiers distinguished good and poor outcomes groups (HR_14GT_ = 3.3) in a validation cohort (n = 254), performing similar to the conventional Clear Code 34 (CC34) RNA expression signature (HR_CC34_ = 2.82)^33^, and providing independent prognostic information in a multivariate Cox regression model of grade and stage (Table S6). To assess the generalizability of the 14VF model we risk stratified cases from the colorectal carcinoma cohort in TCGA. While the *p*-value was significant (*p* = 0.036), the hazard ratio was only 1.65 (data not shown), suggesting the need for retraining our GLMNET models before application in other cancer types.

Other groups have investigated image analysis methods for prognostic biomarker extraction from the tumor microenvironment (TME). Beck *et al*.^11^ quantified H&E stained tissue microarrays of breast cancer and identified distinct stromal features predictive of outcome. Prognostic information was also obtained from the quantification of morphometric heterogeneity of tumor nuclei in glioblastoma multiforme^6,12^. Yuan *et al*.^10^ and Natrajan *et al*.^8^ studied immune invasion and cellular heterogeneity in whole slide images of breast cancers, demonstrating how accurate measurements in histological images can be integrated with genomic data to improve outcomes predictions. A study of ccRCC microvessel hotspots by Sabo *et al*.^22^ used CD34 to quantitate the microvessel density and fractal dimension of microvessels, finding both density and fractal dimension were inversely associated with 5-year recurrence. In contrast to previous image analysis and biomarker studies that analyzed vascular structures in slides stained by immunohistochemistry, our method utilizes an automated EC specific annotation to demarcate vascular structures directly in H&E stained slides. After training on image tiles from only 8 cases, this approach permits immediate analysis of large cohorts of H&E slides without requiring expensive special tissue staining by immunohistochemistry. Further, the approach is not based on the identification and analysis of hotspots of microvessel density, but generates data from $\ge$3 image tiles per case throughout the entire tumor are on the slide.

Our approach of establishing a prognostic RNA based signature based on vascular morphology resulted in the prediction of patient outcomes with a similar accuracy as observed with conventional gene signatures derived through a conventional approach from differentially expressed gene sets. In our morphometric analysis, vascular features related to EC density and vascular arm numbers were higher in good relative to poor outcomes cases (Table 1). We find agreement with the paradoxical observation that higher vascularization density is a favorable trait in ccRCC^34^, possibly explained by uniform blood flow to all parts of the tumor resulting in a lower rate of tumor hypoxia^24^. We also observe higher levels of vascular disorganization, indicated by the standard deviation of vascular arm orientation, in poor, relative to good outcome cases (Table 1). This result suggests ongoing angiogenesis in tumors with a short DFS. In addition, identifying genes that correlate to abstract morphometric features allows biological interpretation of those features. In our case, 8 genes in the 14-gene signature were loosely associated with vascular or heart biology. One gene, SLC16A4 (Tables 2, S5), was previously associated with prognosis in ccRCC. This demonstrates that our unbiased approach led to a gene signature with a connection to vascular biology. However, a detailed analysis of the 180 genes that correlate with expression of the 9 VFs did not reveal any genes in common with the actual hallmark angiogenesis signature of 36 genes^35^. Thus, the 9 VFs may be associated with biological mechanisms connected to a steady state vascular network structure rather than the dynamics of angiogenesis.

While we have demonstrated highly accurate digital image analysis with targeted machine learning methods (EC classification AUC = 0.96), several technical limitations persist. The first limitation arises from hand picking tiles for analysis potentially introducing a sampling error to the analysis which is accentuated by tumor heterogeneity. A natural extension of our approach is to analyze multiple whole slide images per case^36^, ideally serial sections, thus more closely approximating the true distribution of vascular features in 3 dimensions^28^. To accomplish robust whole-slide analysis, the image analysis methods should be updated to use the state-of-the art in convolutional neural networks for computer vision. Multiple steps in vascular quantification would benefit from convolutional neural network methods. Nuclear segmentation and Vascular Area Mask delineation are both tasks well-suited for “semantic segmentation” deep neural networks^37^. A second limitation is the vascular feature palette, composed of targeted features describing the basic shapes and spatial distributions of vascular bodies. Vascular features could be improved by an alternative feature extraction method, such as deep convolutional autoencoders, which would learn features of the vascular network by leveraging the volumes of data in whole slide images. Third, case enrollment based on available data and image quality might introduce a bias in the data. Unbiased machine learning methods require larger cohorts for robust biomarker discovery than do statistical approaches which are based on underlying assumptions. Thus, by using a larger discovery cohort we may increase the generalizability of our vascular biomarkers. This limitation will be addressed in the future by a prospective study. A fourth limitation stems from the sampling error and within tumor heterogeneity affecting correlations between RNA and VF expression. TCGA makes no guarantee that the digital images from pathology slides are from the exact same tissue piece as the RNA sequencing data. Therefore, to account for intratumoral genetic variation, a future study would explore the association between imaging features and genetic sequencing data from carefully collected paired tissue across multiple sites in each tumor.

Altogether, the digital image analysis framework developed for analysis of vascular architecture in H&E slides of ccRCC represents the first attempt to integrate quantitative analysis of tissue structural archetypes with RNA expression data. Through future research, models incorporating morphologic and genomic biomarkers from different platforms will advance the biological understanding of morphologic features, ultimately leading to better patient prognostication and individualized treatments. Pertinent to the treatment of renal cancer, future morphogenomic studies may lead to urgently needed biomarkers predicting response to anti-vascular agents^31,38^.

## Materials and Methods

### Use of experimental human participants

Since images cannot be traced back to patients and do not contain HIPAA sensitive information, or any other information that can lead to patient identification, this research is considered non-subjects research by the institutional review board.

### Image acquisition from slides and immunohistochemistry

Eight H&E slides from local institutional archives were deidentified, anonymized and scanned on an Aperio AT Turbo bright field scanner (Leica Biosystems, 40X magnification, 0.25 micron/pixel). Image tiles of 3,000 pixel^2^ containing only high quality tumor tissue (no folded over or torn tissue, no over- or under-staining), minimal stroma or connective tissue, and without hemorrhage red blood cells were extracted from each slide using Aperio ScanScope software (Leica Biosystems).

H&E slides were decolorized and subsequently stained by immunohistochemistry (IHC) with antibodies reactive to CD31 (V-purple, endothelial cells), and CD45 (DAB, lymphocytes) (Figs S1, S2). The IHC stained slides were digitized on the same slide scanner. Subsequently, image tiles matching those taken from H&E images were extracted from the corresponding IHC slides; the position of each IHC tile was matched to its brother H&E tile by an affine co-registration (MATLAB R2013b, Mathworks, Natick, MA, USA). Hematoxylin images digitally unmixed^25^ from H&E and IHC tiles served as co-registration landmarks. Co-registered H&E and IHC tiles (n = 204) were used for development of image analysis algorithms.

### Hidden Markov Model to process IHC for annotation of H&E images

The following analysis was performed using R. To identify individual cell types, the IHC images were processed by a custom Hidden Markov Model (HMM) classification system, HMMseg. To train the system, pixels of dark brown (DAB), dark purple (CD31), and deep blue (hematoxylin) color were manually collected from regions of IHC stained tissue. To obtain higher quality segmentation, three background states consisting of white (optical background), light blue (cytoplasm), and light brown (residual DAB) were also collected. To analyze IHC stained images, an HMM classifier was trained using the Viterbi algorithm (R package ‘Rhmm’). Ultimately, HMMseg produced binary images demarcating areas of positive IHC staining; these binary masks were regarded as ground truth annotation in the classification pipeline for IHC supervised classification of cell types and tumor vascular areas.

### Cellular classification

Nuclear contours were identified by processing H&E images with the R package ‘CRImage’. In order to increase the stability of this method, the Hematoxylin component of the H&E was color separated, and pre-processed by median filtering (Fig. S3). Features of nuclear morphology and texture were assessed for each individual nucleus.

To construct a dataset for morphological and texture based cellular classification, cellular identities from IHC were imposed upon individual nuclear contours in H&E images. Ground truth for cellular lineages was determined from the CD31 and CD45 masks outputted by HMMseg. This ground truth was dilated and superimposed onto the nuclear segmentations, and labels were imposed on nuclei that had greater than 50% overlap. A training set was gathered consisting of 14,000 cancer, 6,500 endothelial, and 1,500 inflammatory cells from 8 ccRCC slides (Table S1) and used to train a Support Vector Machine classifier.

### Vascular Area Classification

The following analysis was performed using MATLAB R2013b. A second classifier was designed to segment areas of vasculature and to generate vascular area masks (VAM) from H&E images. We observed vascular area as patterns of high eosin stain intensity proximal to endothelial nuclei. To translate the image into features, the locations of endothelial cells and the intensity of eosin staining were used as classification parameters. To leverage these images into a binary representation of vascular area, each pixel in the image tiles was characterized by EC distances (Fig. S5) and eosin intensity values (Fig. S6) in a small surrounding area through a sliding window method. Pixels within the vascular area were marked with reference to the CD31 annotation mask. The binary mask resulting from application of this image processing technique was called the vascular area mask (VAM). VAMs were post-processed, yielding a representation we call the vascular skeleton (VS) and the constituent branch points (BP) and arm images.

### Vascular morphometry features

A set of predetermined binary image features were extracted including object eccentricity, solidity, relative orientations of arms, density, the Euler-Poincare characteristic, the box-counting fractal dimension and sliding-box lacunarity (Fig. S8). Distributions of these image features were collected across all tiles per case. Image features were summarized by the mean, standard deviation, skewness and kurtosis of their distributions, yielding 88 vascular features (VFs) per case.

### Vascular Features predict Disease Free Survival

Clinical data for the TCGA cases was accessed through CBioPortal^39,40^, and H&E whole slide images were downloaded from the TCGA Data Portal. The discovery cohort was composed of 64 cases from 2 institutions which possessed high quality H&E images. From each case, tiles containing maximal tumor area, uninterrupted by tearing, extravasated blood, necrosis, sarcomatoid differentiation or rhabdoid differentiation, was extracted from each whole slide image. To extract the complement of 88 VFs from these tiles we used pre-trained classifiers for endothelial cells and vascular areas. To reduce dimensionality, low variance features (std/mean < 0.3) were excluded. To identify a subset of features with the highest predictive power, a stochastic backwards feature selection method^29,41^ was applied with 1,500 iterations, with each iteration resulting in a set of best features. The results of all iterations were gathered into a final set of 9 VFs. Consensus clustering (R package ‘ConsensusClusterPlus’) was performed on expression levels of 9VFs in each case and average silhouette width (R package ‘factoextra’) confirmed two groups of cases (Fig. S9) which were examined for DFS using a Kaplan-Meier plot.

### Identification of a surrogate gene signature from VFs

The following analysis was performed using R. The VF outcome group classification was further used to train an mRNA expression based classifier. RNA expression data, as reads per kilobase of transcript per million mapped reads (RPKM), was downloaded via FireBrowse (firebrowse.org). We identified a set of 14 genes correlative to the 9 VFs, and with positive Akaike information gain for VF outcome group classification. Two generalized linear models with elastic net regularization (GLMNET) were trained. One model (14VF) was trained on the VF-risk groups and applied to a 301 case validation cohort. The other model (14GT) was trained using 24-months disease free status as the ground truth, and applied to a 252 case validation cohort. Since 5 discovery cases, and 49 validation cohort cases were censored before 24 months, those cases were not included in 14GT training and validation. The DFS prediction by these two models was assessed with Kaplan-Meier plots.

To assess risk group significance in the context of clinical stage (1,2 vs 3,4) and Fuhrman Nuclear Grade (1,2 vs 3,4), a series of multivariate Cox models were trained with differing combinations of bivariate predictors (Table S6). Of the 301 validation cohort cases, 254 also had annotation by a previously reported 34-gene signature (CC34). This overlapping cohort was used to compare the prediction by 14VF and 14GT with prediction by CC34.

The significance of difference between outcome groups was calculated by the Wilcoxon rank-sum test.

### Data availability

Values for the 9VF’s in the discovery cohort, the expression of the 14VF/14GT genes, and the resulting 14VF/14GT classifications in both discovery and validation cohorts, are included as Supplementary Data. Additional data is available by request to the authors.

### Code availability

Source code developed for this work may be accessed through supplementary data files as noted in the text, or by request to the authors.

## Electronic supplementary material

Supplementary Figures and Tables

Supplementary Table 1

Supplementary Table 4

Supplementary Data 1

Supplementary Data 2

Supplementary Data 3
