# Supplementary material for: A novel machine learning approach reveals latent vascular phenotypes predictive of renal cancer outcome
Source: Sci Rep. 2017 Oct 16;7:13190. doi: 10.1038/s41598-017-13196-4 (PMC5643431; doi:10.1038/s41598-017-13196-4)
Supplement: Supplementary file 1 — Supplementary Figures and Tables [file 41598_2017_13196_MOESM1_ESM.pdf]

A novel machine learning approach reveals latent vascular phenotypes predictive of renal cancer outcome

Supplementary Materials

Nathan Ing[1,2], Fangjin Huang[2], Andrew Conley[2], Sungyong You[1], Zhaoxuan Ma[2], Sergey Klimov[2], Chisato Ohe[3], Xiaopu Yuan[2], Mahul B. Amin[3], Robert Figlin[4], Arkadiusz Gertych[1,3*], Beatrice S. Knudsen[2,3,4*]

Affiliations:

[1]Department of Surgery, Cedars Sinai Medical Center, Los Angeles, CA, USA

[2]Department of Biomedical Sciences, Cedars Sinai Medical Center, Los Angeles, CA, USA

[3]Department of Pathology, Cedars Sinai Medical Center, Los Angeles, CA, USA

[4]Samuel Oschin Comprehensive Cancer Institute, Cedars Sinai Medical Center, Los Angeles, CA, USA

*To whom correspondence should be addressed: Arkadiusz.Gertych@cshs.org, Beatrice.Knudsen@cshs.org

Table of Contents

	Page
**Materials and Methods**	3
**Expanded Materials and Methods**	6
**Fig S1.** Classification of endothelial nuclei	11
**Fig S2.** Color classification by Hidden Markov Model to annotate IHC staining in whole slide images	12
**Fig S3.** Example of HMM segmentation results	12
**Fig S4.** Nuclear segmentation from the unmixed hematoxylin image	13
**Fig S5.** Endothelial nuclei location preprocessing for vascular area classification	13
**Fig S6.** Eosin intensity preprocessing for vascular area classification	14
**Fig S7.** Box plot of features for vascular area classification	14
**Fig S8.** Binary vascular object features	15
**Fig S9.** Consensus clustering of discovery cohort cases (n = 64) by 9 VFs	16
**Fig S10.** F-test for inter-case versus intra-case feature variance.	16
**Fig S11.** Hierarchical clustering of cases in the TCGA discovery cohort by 9VFs into good (blue) and poor (red) outcomes groups	17
**Fig S12.** Heat map of expression values of the 14GC in the discovery cohort	18
**Fig S13.** Visualization of expression levels of the 14GC in the discovery cohort	19
**Fig S14.** Comparison of the 6 prediction models fit with the 14GC to separate good and poor prognosis groups – as determined by the 9 VFs – in the discovery cohort	19
**Fig S15.** Kaplan-Meier plot of 14GC good and poor prognosis groups in the discovery cohort	20
**Fig S16.** Visualization of expression levels for the 14GC in the validation cohort (n = 301)	20
**Table S1.** List of nuclear morphology and texture features from CRImage	Excel
**Table S2.** Training and validation set metrics for nuclear and vascular area classifiers	21
**Table S3.** Clinical variables for patient cohorts	22
**Table S4.** 182 unique genes correlated with vascular features	Excel
**Table S5.** Functional annotation of genes in the 14 gene classifier	23
**Table S6.** Multivariate Cox regression models for disease free survival	24
**Table S7.** Description of 9 VFs	25
**Data File S1.** Demonstration code for Hidden Markov Model segmentation in IHC images	26
**Data File S2.** Demonstration code for Vascular Area Mask segmentation from H&E images.	27
**Data File S3.** Vascular features and disease free survival groups of cases in the TCGA discovery and validation cohorts	Excel
**Supplementary References**	26

Materials and Methods

Image acquisition from slides and immunohistochemistry

Eight H&E slides from local institutional archives were deidentified, annonymized and scanned on an Aperio AT Turbo bright field scanner (Leica Biosystems, 40X magnification, 0.25 micron/pixel). Since images cannot be traced back to patients and do not contain HIPAA sensitive information or any other information that can lead to patient identification, this research is considered non-subjects research by the institutional review board. Image tiles of 3,000 pixel^2 containing only high quality tumor tissue (no folded over or torn tissue, no over- or under-staining), minimal stroma or connective tissue, and without hemorrhage red blood cells were extracted from each slide using Aperio ScanScope software (Leica Biosystems).

H&E slides were decolorized and subsequently stained by immunohistochemistry (IHC) with antibodies reactive to CD31 (V-purple, endothelial cells), and CD45 (DAB, lymphocytes) (**Fig. S1, S2**). The IHC stained slides were digitized on the same slide scanner. Subsequently, image tiles matching those taken from H&E images were extracted from the corresponding IHC slides; the position of each IHC tile was matched to its brother H&E tile by an affine co-registration (MATLAB R2013b, Mathworks, Natick, MA, USA). Hematoxylin images digitally unmixed [22] from H&E and IHC tiles served as co-registration landmarks. Co-registered H&E and IHC tiles (n = 204) were used for development of image analysis algorithms.

Hidden Markov Model to process IHC for annotation of H&E images

The following analysis was performed using R. To identify individual cell types, the IHC images were processed by a custom Hidden Markov Model (HMM) classification system, HMMseg. To train the system, pixels of dark brown (DAB), dark purple (CD31), and deep blue (hematoxylin) color were manually collected from regions of IHC stained tissue. To obtain higher quality segmentation, three background states consisting of white (optical background), light blue (cytoplasm), and light brown (residual DAB) were also collected. To analyze IHC stained images, an HMM classifier was trained using the Viterbi algorithm (R package 'Rhmm'). Ultimately, HMMseg produced binary images demarcating areas of positive IHC staining; these binary masks were regarded as ground truth annotation in the classification pipeline for IHC supervised classification of cell types and tumor vascular areas.

Cellular classification

Nuclear contours were identified by processing H&E images with the R package 'CRImage'. In order to increase the stability of this method, the Hematoxylin component of the H&E was color separated, and pre-processed by median filtering (**Fig. S3**). Features of nuclear morphology and texture were assessed for each individual nucleus.

To construct a dataset for morphological and texture based cellular classification, cellular identities from IHC were imposed upon individual nuclear contours in H&E images. Ground truth for cellular lineages was determined from the CD31 and CD45 masks outputted by HMMseg. This ground truth was dilated and superimposed onto the nuclear segmentations, and labels were imposed on nuclei that had greater than 50% overlap. A training set was gathered consisting of 14,000 cancer, 6,500 endothelial, and 1,500 inflammatory cells from 8 ccRCC slides (**Table S1**) and used to train a Support Vector Machine classifier.

Vascular Area Classification

The following analysis was performed using MATLAB R2013b. A second classifier was designed to segment areas of vasculature and to generate vascular area masks (VAM) from H&E images. We observed vascular area as patterns of high eosin stain intensity proximal to endothelial nuclei. To translate the image into features, the locations of endothelial cells and the intensity of eosin staining were used as classification parameters. To leverage these images into a binary representation of vascular area, each pixel in the image tiles was characterized by EC distances (**Fig. S5**) and eosin intensity values (**Fig. S6**) in a small surrounding area through a sliding window method. Pixels within the vascular area were marked with reference to the CD31 annotation mask. The binary mask resulting from application of this image processing technique was called the vascular area mask (VAM). VAMs were post-processed, yielding a representation we call the vascular skeleton (VS) and the constituent branch points (BP) and arm images.

Vascular morphometry features

A set of predetermined binary image features were extracted including object eccentricity, solidity, relative orientations of arms, density, the Euler-Poincare characteristic, the box-counting fractal dimension and sliding-box lacunarity (**Fig. S8**). Distributions of these image features were collected across all tiles per case. Image features were summarized by the mean, standard deviation, skewness and kurtosis of their distributions, yielding 88 vascular features (VFs) per case.

Vascular Features predict Disease Free Survival

Clinical data for the TCGA cases was accessed through CBioPortal [1,2], and H&E whole slide images were downloaded from the TCGA Data Portal. The discovery cohort was composed of 64 cases from 2 institutions which possessed high quality H&E images. From each case, tiles containing maximal tumor area, uninterrupted by tearing, extravasated blood, necrosis, sarcomatoid differentiation or rhabdoid differentiation, was extracted from each whole slide image. To extract the complement of 88 VFs from these tiles we used pre-trained classifiers for endothelial cells and vascular areas. To reduce dimensionality, low variance features (std/mean < 0.3) were excluded. To identify a subset of features with the highest predictive power, a stochastic backwards feature selection method [3] was applied with 1,500 iterations, with each iteration resulting in a set of best features. The results of all iterations were gathered into a final set of 9 VFs. Consensus clustering (R package 'ConsensusClusterPlus') was performed on expression levels of 9VFs in each case and average silhouette width (R package 'factoextra') confirmed two groups of cases (**Fig. S9**) which were examined for DFS using a Kaplan-Meier plot.

Identification of a surrogate gene signature from VFs

The following analysis was performed using R. The VF outcome group classification was further used to train an mRNA expression based classifier. RNA expression data, as reads per kilobase of transcript per million mapped reads (RPKM), was downloaded via FireBrowse

(http://firebrowse.org). We identified a set of 14 genes correlative to the 9 VFs, and with positive Akaike information gain for VF outcome group classification. Two generalized linear models with elastic net regularization (GLMNET) were trained. One model (14VF) was trained on the VF-risk groups and applied to a 301 case validation cohort. The other model (14GT) was trained using 24-months disease free status as the ground truth, and applied to a 252 case validation cohort. Since 5 discovery cases, and 49 validation cohort cases were censored before 24 months, those cases were not included in 14GT training and validation. The DFS prediction by these two models was assessed with Kaplan-Meier plots.

To assess risk group significance in the context of clinical stage (1,2 vs 3,4) and Fuhrman Nuclear Grade (1,2 vs 3,4), a series of multivariate Cox models were trained with differing combinations of bivariate predictors (**Table S6**). Of the 301 validation cohort cases, 254 also had annotation by a previously reported 34-gene signature (CC34). This overlapping cohort was used to compare the prediction by 14VF and 14GT with prediction by CC34.

The significance of difference between outcome groups was calculated by the Wilcoxon rank-sum test.

Code Availability

Source code developed for this work may be accessed through supplementary data files as noted in the text, or by request to the authors.

Expanded Materials & Methods

Image acquisition

Eight H&E slides from local institutional archives were scanned by an Aperio AT Turbo bright field scanner (Leica Biosystems, 40X magnification, 0.25 micron/pixel) (**Fig. S1**). H&E slides were decolorized and subsequently stained by immunohistochemistry (IHC) with antibodies reactive to CD31 (V-purple, endothelial cells), and CD45 (DAB, lymphocytes). The IHC stained slides were digitized on the same slide scanner.

Information on the scanner make and models used at individual TCGA-contributing sites was unavailable. Whole slide image files in SVS format were downloaded through the TCGA data portal.

Immunohistochemistry

Antibodies were used in the sequence of CD45 → CD31 for staining of the same tissue section. For CD45, antigen retrieval occurred with Na/EDTA pH 8.0 for ~30 minutes @ 90˚C. Tissues were blocked with animal-free protein blocking buffer (Vector Laboratories cat. # SP-5030) for 15 minutes. To quench the endogenous peroxide, the tissue was treated with H_2O_2 for 12 minutes. The anti-CD45 (Ventana Pre-Dilute, cat. # 790-2505) was applied for ~30 minutes @ 37˚C in the DISCOVERY ULTRA automated slide stainer (Ventana cat. # 750-601). Thereafter, the EnVision+ System – HRP labeled polymer goat anti-mouse secondary antibody (Dako cat. # K400011) was used for 20 minutes, followed by DAB (3,3'-diaminobenzidine, Vector Laboratories cat. # SK-4100) staining for 8 minutes.

Next, slides were incubated with the denaturing buffer (citrate buffer pH 6) for 10 minutes @ 110˚C, to remove the CD45 antibody. Tissues were blocked with animal-free protein blocking buffer (Vector Laboratories cat. # SP-5030) for 16 minutes. To quench the endogenous peroxide, the tissue was treated with H_2O_2 for 12 minutes. The CD31 antibody (Cell Signaling, cat # 3528) was diluted at 1:1000 and applied for ~60 minutes @ 37˚C in the DISCOVERY ULTRA automated slide stainer (Ventana cat. # 750-601). Thereafter, the EnVision+ System – HRP labeled polymer goat anti-mouse secondary antibody (Dako cat. # K400011) was used for 32 minutes. DISCOVERY Purple (Ventana, cat. # 253-4857) was applied as the chromogen to visualize CD31-antibody binding for 24 minutes. Slides were stained with Modified Mayer's Hematoxylin (American MasterTech Scientific, cat. # HXMMPT) for 1.5 minutes and cover-slipped.

Annotation of IHC images by Hidden Markov Model

IHC images were annotated for areas positive for CD31, CD45, or hematoxylin by an in-house Hidden Markov Model (HMM) classification system, HMMseg. Blank areas on slides were also annotated. HMMseg utilizes HMM series prediction ('Rhmm') [4] and Support Vector Machine (SVM; 'e1071') [5] classification to detect areas positive for each stain. To train the system, pixels of dark brown (DAB), dark purple (CD31), and deep blue (hematoxylin) color were manually collected from example regions of IHC stained tissue. To obtain higher quality segmentation, three background states consisting of white (optical background), light blue (cytoplasm), and light brown (residual DAB) were similarly collected. (In all 15,000 training pixels were collected.) To obtain the precursor probability and transition matrices for the HMM, a multi-class SVM was trained using the six colors, then applied to a small representative image

tile, thereby obtaining an estimate for proportion of each color. An HMM classifier was trained using the Viterbi algorithm ('Rhmm'). The Viterbi algorithm predicted the state transitions for a vector of pixels in one dimension: across all rows or columns of the image. This resulted in two solutions per image. These solutions were compared, and points of disagreement were resolved by the previously trained SVM classifier. Each annotation that represented a positive stain was independently post processed to fill small holes (area < 75 pixels) and to remove small regions (area < 75 pixel). Boundaries were smoothed by morphological opening (diamond structuring element with a 3-pixel radius; 'EBImage') [6]. Ultimately, HMMseg produced binary images demarcating areas of positive IHC staining (**Fig. S2**); these binary masks were regarded as ground truth annotation in the following classification pipeline for IHC supervised classification of cell types and tumor vascular areas.

Automated annotation of segmented nuclei for training a cellular classifier

A first classifier was trained to identify nuclei of different lineages in H&E images by their nuclear morphology and texture. Hematoxylin images were color-unmixed from H&E images and then preprocessed by a median filter to fill nuclear interiors and suppress background noise. Nuclei were then segmented by a seeded-watershed technique ('CRImage'; maxShape = 300, minShape = 200, failureRegion = 7000, medianFilter = TRUE, edgeDetection = TRUE, speckleSize = 150, watershedTolerance = 0.9, postEdgeFill = TRUE, whiteHigh = 0.85, normalize = TRUE, numWindows = 20) (**Fig. S1; Fig. S4**), and each nucleus was parameterized by 63 features of morphology and texture ('CRImage'; **Table S1**). Ground truth lineage of nuclei was determined from the CD31 and CD45 masks outputted by HMMseg. Each mask was first processed by morphological dilation (disk, radius of 11 pixels), then overlaid onto the mask with parametrized nuclear contours. Nuclei that had greater than 50% overlap were assigned to a specific lineage. By this method, 14,000 cancer, 6,500 endothelial, and 1,500 inflammatory cells from 8 locally archived ccRCC slides (**Table S3**) were labeled with reference to IHC annotation and included for classifier training. We used the same SVM classification method as in Yuan, et al. (2012) to perform nuclear classification using these labelled nuclei. To compensate for uneven class representation in the training set, the SVM (radial kernel, g = 0.0159) was created with retrospectively determined weights (tumor = 0.9, endothelial = 1.3, lymphocyte = 2). Accuracy of the classifier was assessed with a test set of 255,000 nuclei, notably with similar proportions to the training set (**Table S3**). This classifier was applied to classify de novo nuclei into the three classes of cellular lineage.

Pixel-wise classification to delineate vascular areas in H&E images

A second classifier was designed to segment areas of vasculature. The locations of endothelial cells and the intensity of eosin staining were used as classification parameters. The eosin image component was unmixed from H&E images and then normalized by histogram normalization. Sequential application of anisotropic diffusion filter [7] (25 iterations, edge threshold 20, Δt = 0.2, σ = 0.5), Sobel filter, image reconstruction and averaging filter (radius = 7) produced a smoothed image wherein eosinophilic areas were enhanced - made to be uniformly dark (**Fig. S6**). A distance transform on the binary mask of endothelial nuclei provided a numerical representation of distance between EC (**Fig. S5**). The distance transform image and the enhanced eosin image were used to find pixels of vascular tree through a pixel-wise classification.

To leverage these images into a vascular area representation, each pixel was characterized by EC distances and eosin intensity values in a small surrounding area from image tiles. Pixels of vasculature were marked with reference to the CD31 annotation mask. The mask was overlaid on the eosin intensity and EC distance transform images. Random Forrest classifiers were previously used by Gertych, et al. (2015) in a similar histopathological image segmentation, with analogous features. Image features of 6,000 pixels under the mask were extracted and used to train a Random Forrest classifier (MATLATB R2014b, Statistics & Machine Learning Toolbox). The classifier was then validated on approximately n = 5.0e10, test pixels from n = 210 tiles (**Table S3**). The mask resulting from application of this classification technique, in each image tile, was called the vascular area mask (VAM; **Fig. 1B**). VAMs were post-processed by small hole-filling (< 200 px), short bridge connection (iterative closings with a rotating linear structuring element, 15-pixel length) and midline transformation yielding a representation we call the vascular skeleton (VS). This VS consists of many intersecting single-pixel width splines (**Fig. 1D**). In the VS, points of intersection were defined as branching points (BP), and vascular arms were obtained by subtracting the BP from the VS. Together, the VAM, VS, BP and EC masks were used as the basis for characterization of the tumor vasculature.

Vascular Feature extraction

A set of predetermined binary image features were extracted including object eccentricity, solidity, relative orientations of arms, density (MATLAB, "regionprops" function), the Euler-Poincare characteristic, the box-counting fractal dimension and sliding-box lacunarity (**Fig. S8**). Features were collected as distributions across all tiles per case in the following way. Let $\mathbf{T}$ be the set of all tiles in a single case, and $\mathbf{O}$ be all binary objects (i.e. endothelial cells) in a tile. Distribution $\boldsymbol{D_f}$ is composed of feature values of feature f, for all $\mathbf{O}$, in all $\mathbf{T}$. In the case of features calculated on whole tiles, like fractal dimensions, the distribution $\boldsymbol{D_f}$ is simply composed of values $\boldsymbol{f}$ for all tiles $\mathbf{T}$. Each $\boldsymbol{D_f}$ was then summarized by its mean, standard deviation, skewness and kurtosis, also known as the histogram moments. All together each case was described by 88 vascular features (VF's). The range of $\boldsymbol{N}$ was from 3 to 74 tiles per case ($\mu \approx 25$, $\sigma \approx 18$) in the 64 case TCGA discovery cohort.

Analysis of features for disease free survival

We sought to assess VFs in the context of disease free survival (DFS) prediction. Clinical data for the TCGA cases was accessed through CBioPortal. Of the 537 ccRCC cases in the Cancer Genome Atlas (TCGA), 64 cases were selected that had both sufficient image quality, and complete disease free survival data (**Table S2**). Images were assessed visually using Aperio ScanScope (Leica Biosystems) software. Image tiles were selected that possessed uninterrupted tumor area of >9,000 px^2 (at least 3 * 3,000 px^2 tiles), < 10% area covered by extravasated red blood cells, near 0% necrosis, sarcomatoid, or rhabdoid differentiation. Additionally, cases with weak hematoxylin and eosin staining were excluded. The maximum area per case that fit the above criteria were gathered as tiles for VF extraction.

The full complement of 88 VF's were extracted from the 64 discovery cohort cases. To reduce dimensionality, low variance features (std/mean < 0.3) were excluded. To identify a subset of features with the highest predictive power, a backwards feature selection method was applied

with 1,500 iterations. Briefly, we first identified a baseline log-rank p-value by clustering (average correlation distance) a random 75% subset of the 64 cases into two groups by their expression of all VF's. The resulting DFS curves were analyzed for significance by the Kaplan-Meier method. Iteratively, the cases were re-clustered after removal of one VF. VF's were excluded according to the most improvement in log-rank test p-value at each step. This process repeated until removal of additional VF's would result in an increased p-value, i.e. worse separation. At this point, the remaining VF's were tracked, and the process began again with the full VF complement. The remaining VF's, from 1,500 such iterations, were tallied, and the top 9 most frequently selected VF's were selected for further analysis. Consensus clustering ('ConsensusClusterPlus' package) was performed on these 9 features and average silhouette width ('factoextra' package) confirmed two groups of cases (**Fig. S9**) which were examined for DFS by Kaplan-Meier analysis. Subsequently, the remaining 301 TCGA ccRCC samples were evaluated for image quality. Of the slide images available, 28 were of sufficient quality for VF analysis. To classify the 28 cases into good and poor outcome groups, we trained a Random Forest classifier (MATLAB, 30 trees, other settings default) on the VF signature from the 64 case discovery cohort.

Identification of a surrogate gene signature from VF's

The following analysis was performed using R version 3.3 [8]. Samples from the discovery set were assigned into 2 groups by hierarchical clustering using correlation distance and average agglomeration, with values of the 9 selected vascular image features (VF). This VF group classification was further used to train mRNA expression based classifier. RNA expression data, as reads per kilobase of transcript per million mapped reads (RPKM), was downloaded via FireBrowse (http://firebrowse.org). RPKM data was preprocessed by log-2 transform and quantile normalized. We chose a small subset of genes that are highly correlated (top 0.05 percentile Pearson's correlation coefficient) with each of the 9 selected image features. Thus a 182 gene set was obtained (**Table S3**). As in Yu & Snyder, et al. (2016), the gene set was further refined by calculating the information gain ratio for each gene ('FSelector' package) [9]. Fourteen genes were identified with positive information gain and these were included in the final gene set (**Table S5**).

The VF outcome group classification was further used to train an mRNA expression based classifier. RNA expression data, as reads per kilobase of transcript per million mapped reads (RPKM), was downloaded via FireBrowse (http://firebrowse.org). We identified a set of 14 genes correlative to the 9 VFs, and with positive Akaike information gain for VF outcome group classification. Two generalized linear models with elastic net regularization (GLMNET) were trained. One model (14VF) was trained on the VF-risk groups and applied to a 301 case validation cohort. The other model (14GT) was trained using 24-months disease free status as the ground truth, and applied to a 252 case validation cohort. Since 5 discovery cases, and 49 validation cohort cases were censored before 24 months, those cases were not included in 14GT training and validation. The DFS prediction by these two models was assessed with Kaplan-Meier plots.

To assess risk group significance in the context of clinical stage (1,2 vs 3,4) and Fuhrman Nuclear Grade (1,2 vs 3,4), a series of multivariate Cox models were trained with differing combinations of bivariate predictors (**Table S6**).

The significance of difference between outcome groups was calculated by the Wilcoxon rank-sum test.

Supplementary Figures

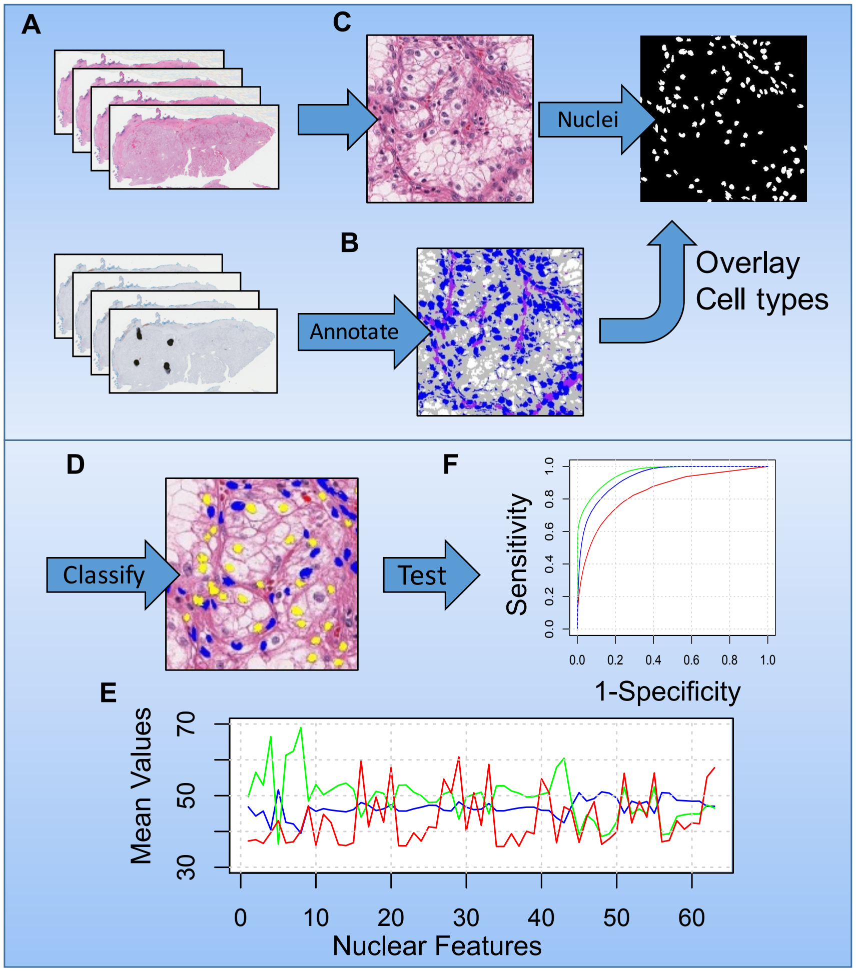

Fig. S1. ***Classification of endothelial nuclei*****. (A) Slides for classifier training.** The top group shows H&E stained slides and the bottom group shows the exact same slides stained by immunohistochemistry with antibodies reactive with CD31 and CD45. **(B+C)** ***Integration of H&E and IHC data for classifier training.*** **(B)** *Hidden Markov model:* Visualization of results from hidden Markov model applied to IHC tile images, CD31 – purple, Hematoxylin – blue, CD45 (not shown) – red). **(C)** *Nuclear mask:* Nuclear contours as delineated from the hematoxylin portion of the H&E image. **(D-F) Classifier testing (F)** *Example of cellular classification result:* endothelial nuclei – blue, tumor nuclei – yellow, lymphocytes (not shown) – red. **(E)** *Expression levels of extracted features used in the classification:* Average expression (y-axis) of nuclear features (x-axis) separating endothelial cells (green), tumor cells (red) and lymphocytes (blue). **(F)** *Classifier performance:* Cellular classification accuracy assessed by receiver operating characteristic curves in a testing set of 255,000 nuclei annotated through immunohistochemistry (AUC: tumor - blue = 0.93, endothelial cells - green = 0.96, lymphocytes - red = 0.84).

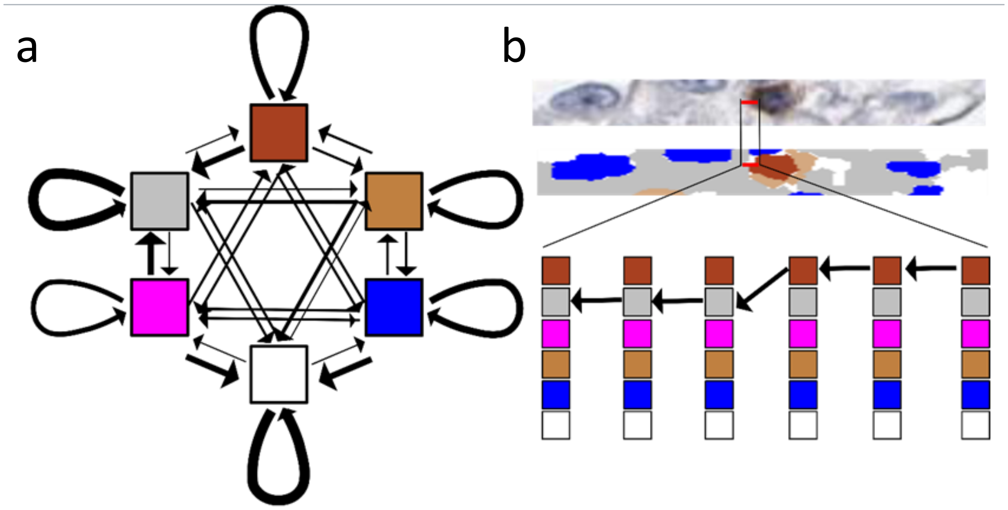

Fig. S2. ***Color classification by Hidden Markov Model (HMM) to annotate IHC staining in whole slide images.*** **(a)** Six states were identified representing three stains (hematoxylin (blue), CD31 (purple), CD45 (brown)) and three background colors (white, grey and light brown). These states have a certain probability of transitioning between states, indicated by arrow weight. For example, it is very likely to transition from blue to white, and from white back to white. **(b)** A series of these states were predicted for each row and column in an image. Traversal of image data as whole rows and columns reduces the rate of single pixels, e.g. within a nucleus, with open chromatin conformation, being misclassified.

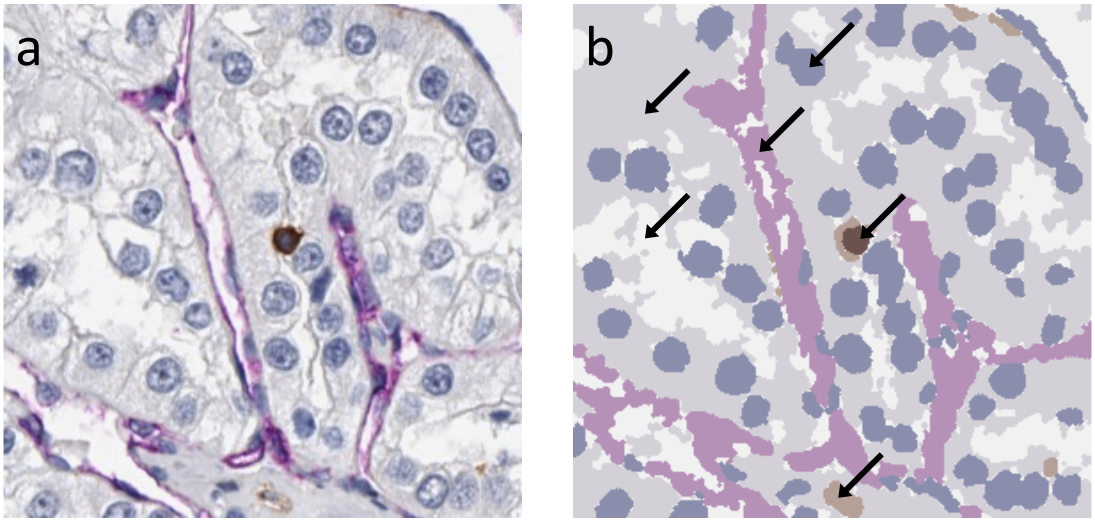

Fig. S3. ***Example of HMM segmentation results.*** **(a)** Original image of CD31 (purple) and CD45 (brown) stained tissue. Blue hematoxylin marked DNA in tumor cell nuclei. **(b)** HMMseg output image. Each pixel was assigned to one of six classes, and artificially colored for visualization. All six colors (blue, grey, light brown, purple, brown and white) are visible in this example (arrows). This model was trained with ~15,000 training pixels taken from six small (~200 sq. px.) regions, and the posterior probabilities determined by SVM classification of an image 300 x 300 pixels large.

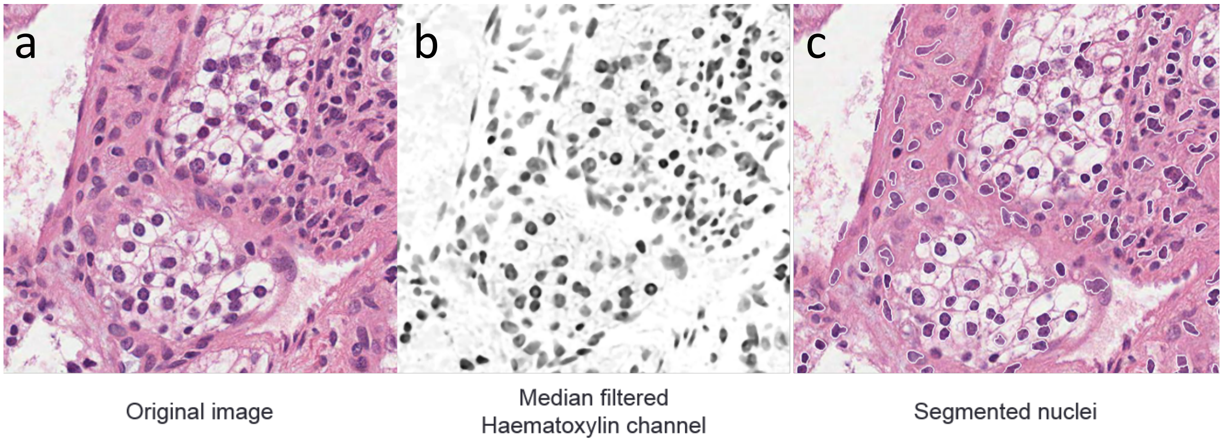

Fig S4. ***Nuclear segmentation from the unmixed hematoxylin image.*** **(a)** Original image of hematoxylin and eosin (H&E) stained tissue. **(b)** The hematoxylin stain component was isolated by numerical deconvolution, followed by median filtering to smoothen unevenly stained nuclei. Pre-processing reduced the frequency of under-segmentation of nuclei, and increased the quality of segmentation faithfully outlining the contour of each nucleus. **(c)** Nuclear contours, delineated by the 'CRImage' package.

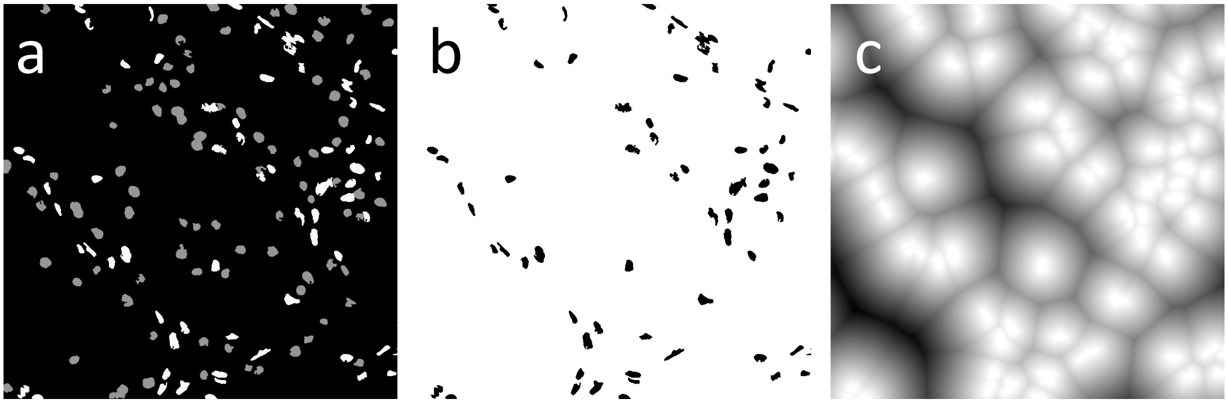

Fig. S5. ***Endothelial nuclei location preprocessing for vascular area classification.*** **(a)** All nuclei (endothelial, lymphocyte, and tumor) in the image are represented as a label image after classification by the cellular classifier. **(b)** Nuclei classified as endothelial cells were isolated from other nuclei. In this image, each dark spot represents an endothelial nucleus. **(c)** A distance transformation was applied to the image, encoding the distance from the nearest endothelial nucleus for all pixels. Dark represents a greater distance. For example, the blue arrows in (b) and (c) point at the same area. The image (c) is colored white to represent the presence, and proximity, of the endothelial nuclei. The thin gray lines throughout (c) are local maxima, and occur at the midpoint between neighboring nuclei. This pipeline provides information on the location of endothelial nuclei as a part of the vascular area classifier.

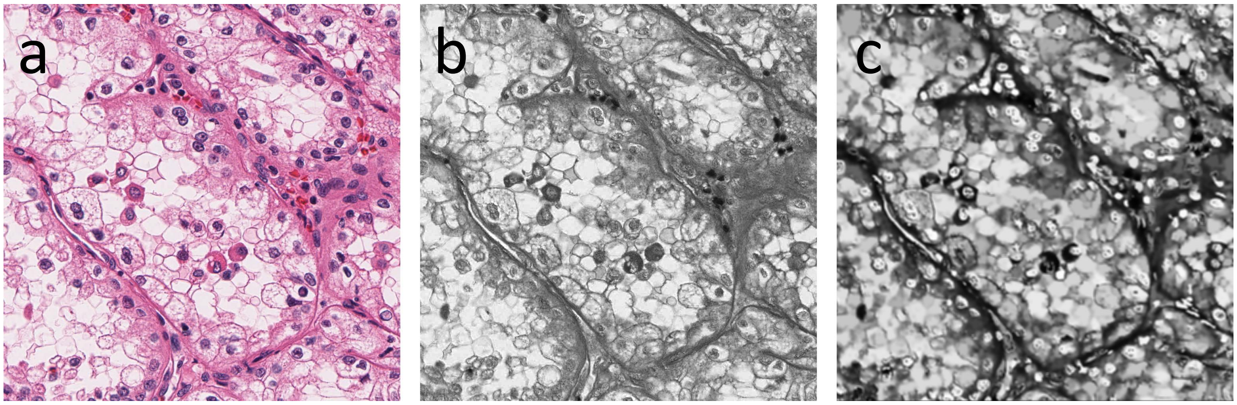

Fig. S6. ***Eosin intensity preprocessing for vascular area classification.*** **(a)** Original H&E image. **(b)** Color separated eosin stain intensity. Color deconvolution to isolate eosin intensity was performed by the same method as in nuclear segmentation. Target mean and standard deviation matrices were set as constants for all experiments. **(c)** Processed eosin image by hematoxylin subtraction, anisotropic diffusion filtering, sobel filtering, image reconstruction, and average filtering. See online methods for individual filter parameters. This pipeline provides information of perivascular tissue as a part of the vascular area classifier.

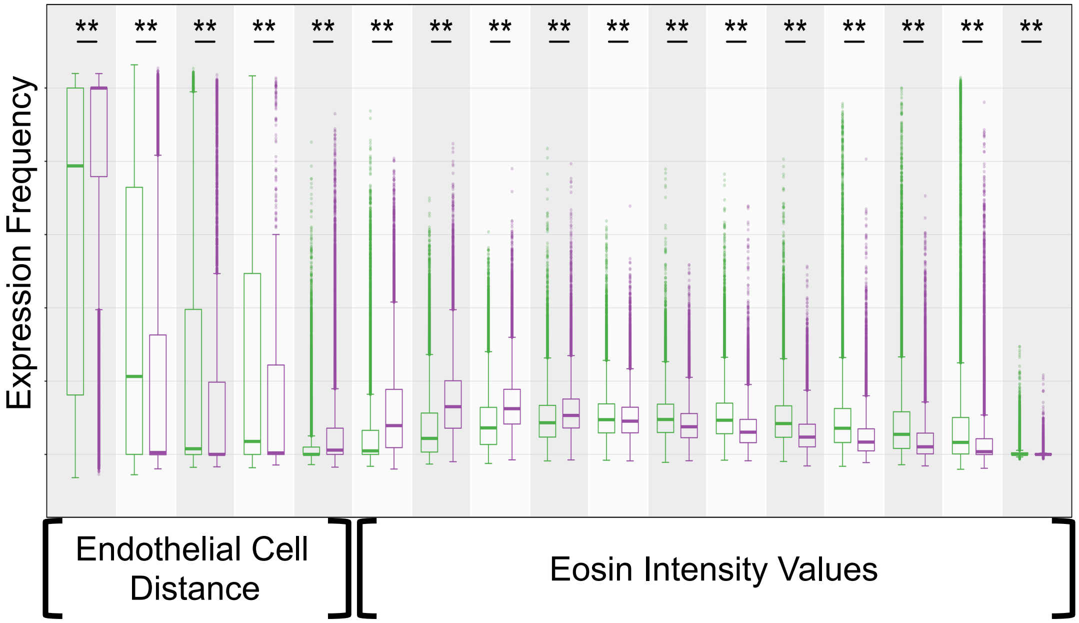

Fig. S7. ***Box plot of features for vascular area classification***. Features along the x-axis are bins of local intensity histograms from the endothelial cell distance image (**Fig. S5**) and the eosin intensity image (**Fig. S6**). The points centered over CD31-positive or CD31-negative areas, as defined by HMM annotation, are colored purple and green respectively. Values are ordered low to high from left to right. For example, low value of endothelial cell distance is significantly more expressed for CD31-positive areas, indicating close proximity of EC to the vasculature. Wilcoxon rank-sum test was used to test statistical difference between the two groups, ** = $p <$ 0.01.

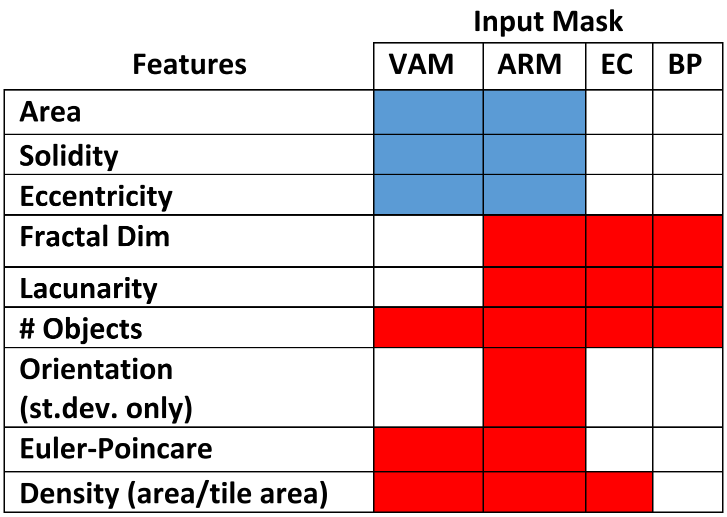

Fig. S8. ***Binary vascular object features.*** **Red** denotes a parameter which is obtained from analyzing the whole image tile. These parameters measure the objects in the tile. **Blue** denotes a parameter that is obtained for each object in an image. **White** marks a mask and feature combination that was not included. The total of Red plus Blue is 22 image analysis features. Multiple values are obtained for each feature and plotted as a distribution. Each distribution is characterized by 4 values (mean, STD, skewness and kurtosis), generating 88 VFs to numerically capture the organization of the vasculature.

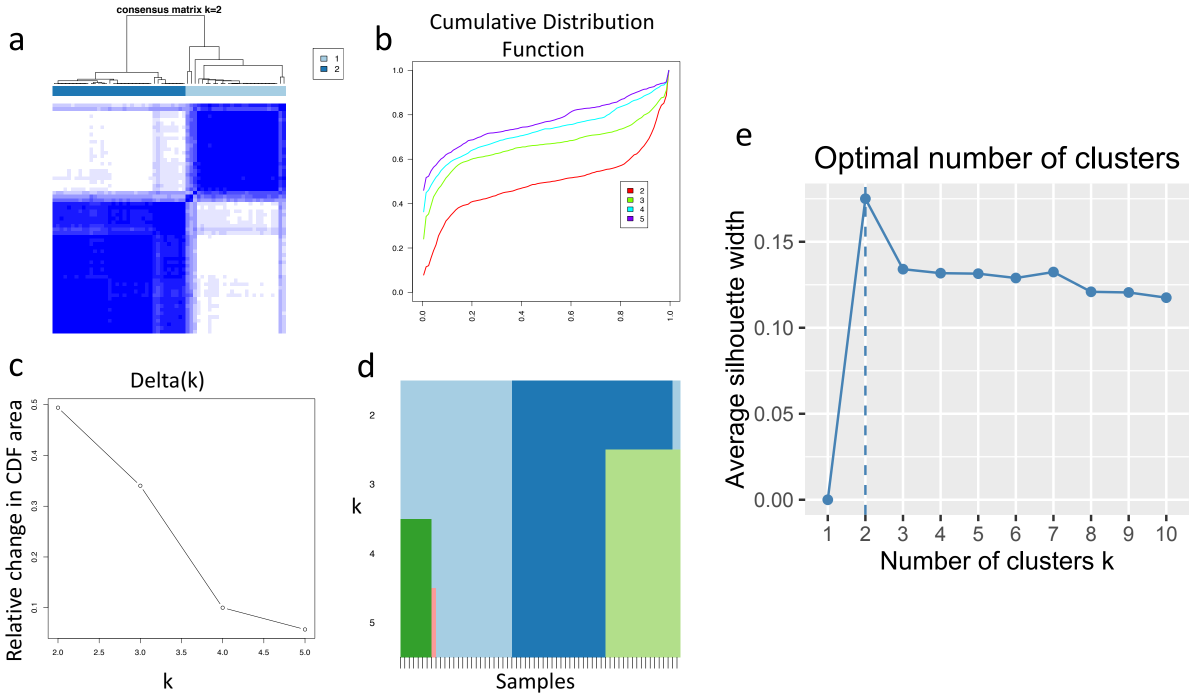

Fig. S9. *Consensus clustering of discovery cohort cases (n = 64) by 9 VFs.* **(a)** Consensus matrix for k=2 clusters showing the frequency of co-clustering for each case over 100 iterations. **(b)** Cumulative distribution functions (CDF) for k = 2,3,4,5. **(c)** Change in the area under the CDF. The largest change observed with the transition from k=1 to k=2, with a still-significant increase with the transition from k=2 to k=3, and marked levelling off for k>3. **(d)** Tracking plot shows the consensus group assignments for each case (columns) as the number of clusters, k, increases. **(e)** Average silhouette width over 100 iterations demonstrates that 2 clusters optimally encompass the data.

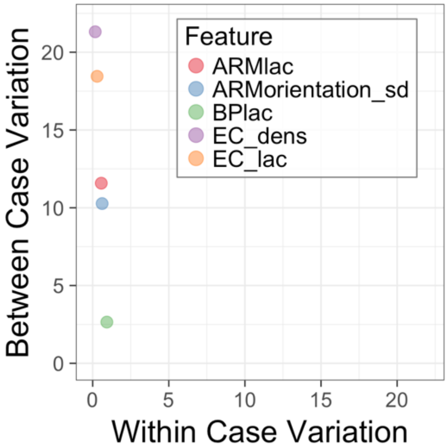

Fig. S10. F-test for inter-case versus intra-case feature variance. The features found important for survival prediction were tested for variance between the 64 discovery cohort cases, compared with the variance between tiles from the same cases. All features vary significantly more between cases than within case ($p < 0.001$).

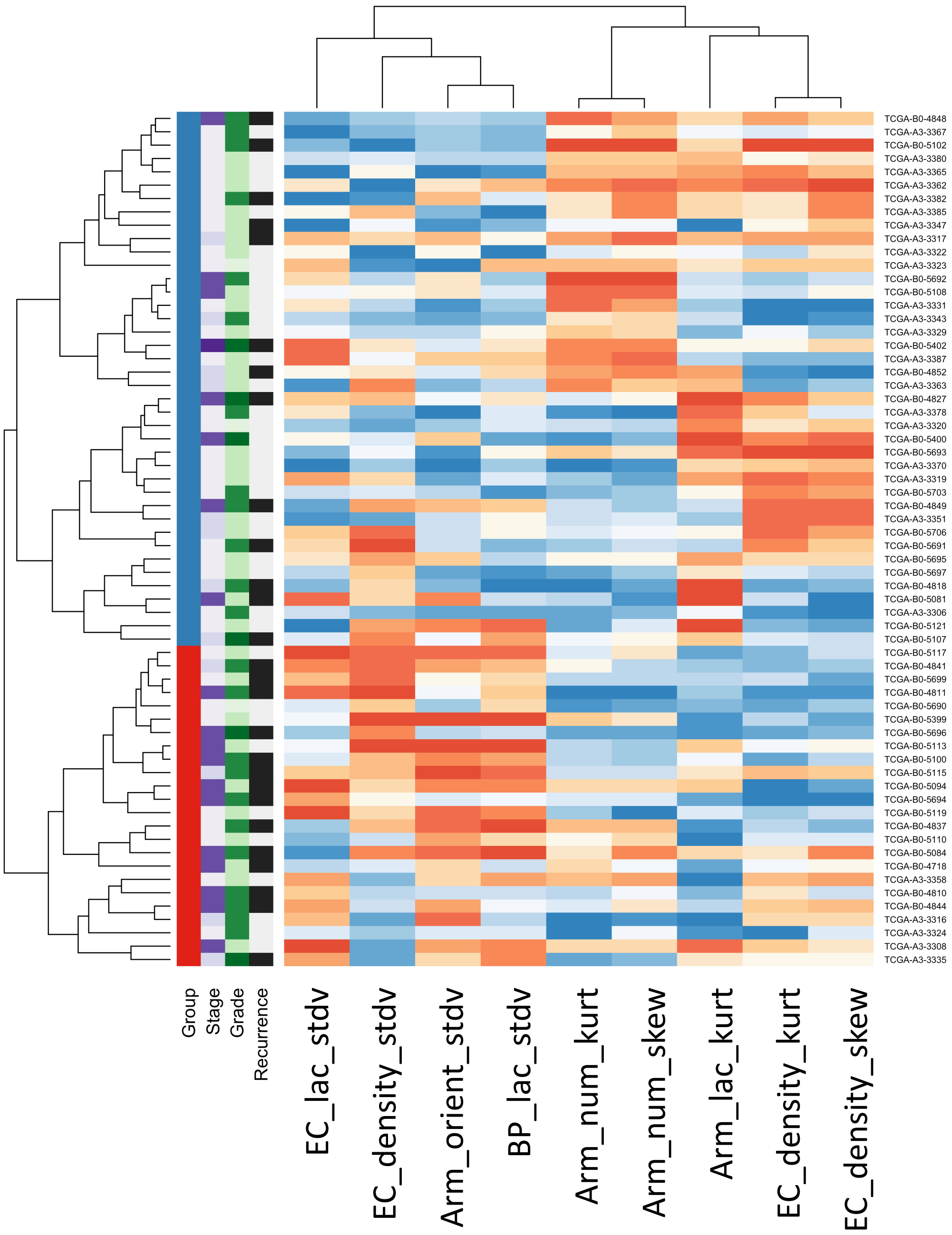

Fig. S11. ***Hierarchical clustering of cases in the TCGA discovery cohort by 9 VFs into good (blue) and poor (red) outcomes groups.*** The heatmap is the same as shown in **Fig. 2B** with annotation of VFs below each column. The stage, Furman grade and recurrence status are indicated on the left of each case. See **Table 2** for a short interpretation of each vascular feature.

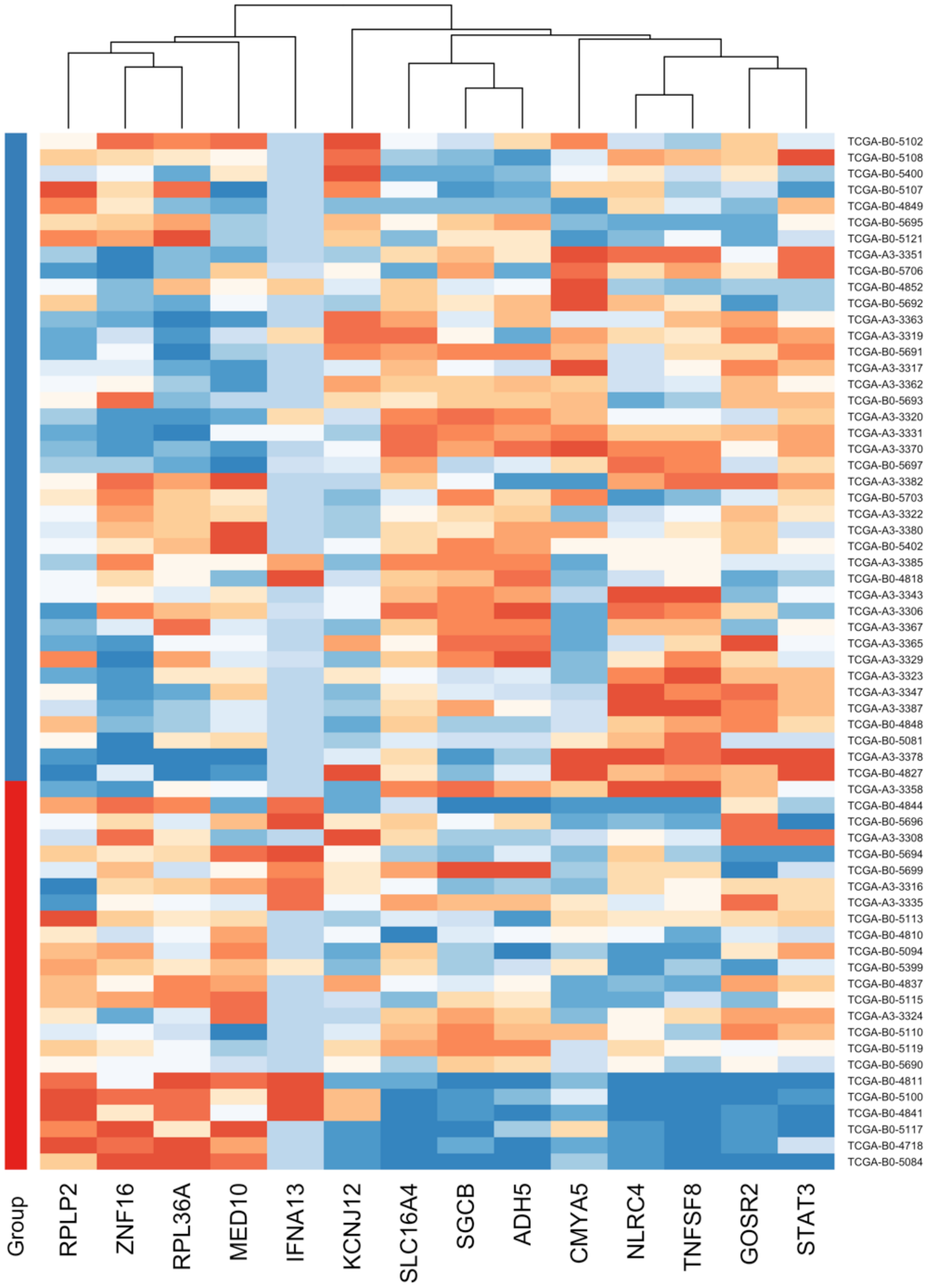

Fig. S12. ***Heat map of expression values of the 14GC in the discovery cohort (n=64).*** Shades of red indicate high expression, and shades of blue indicate low expression after median centering. The red and blue label bar indicates cases assigned to good (blue) and poor (red) outcome groups by VF clustering. The sample rows in this heat map were ordered according to the outcomes prediction obtained with the 9 VF classifier. Columns were arranged by hierarchical clustering (correlation distance).

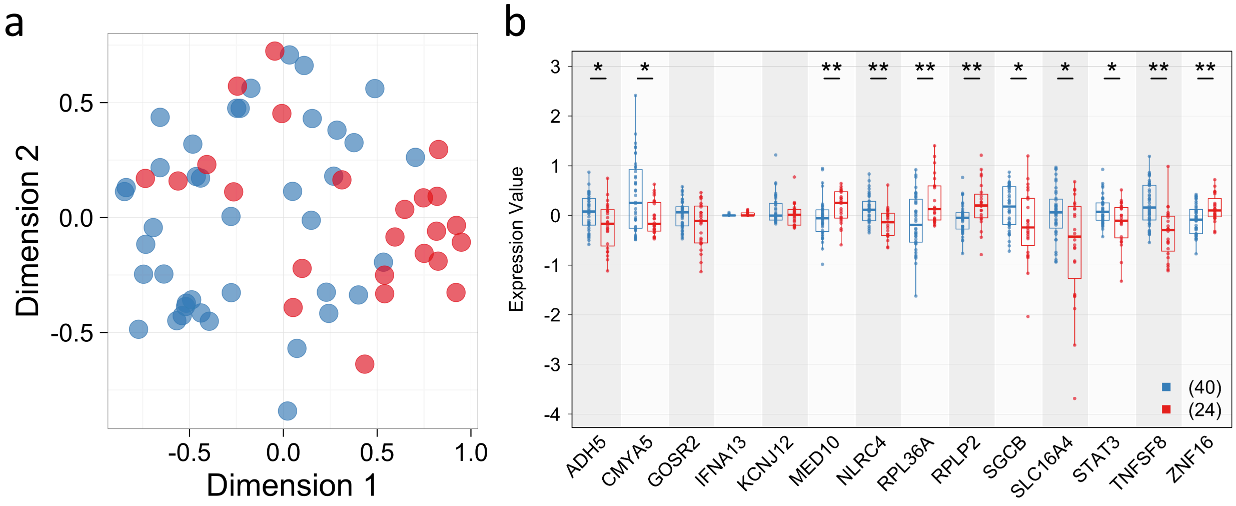

Fig. S13. ***Visualization of expression levels of the 14GC in the discovery cohort (n = 64).*** **(a)** Multidimensional scaling plot (average correlation distance) of 9 VF depicting good (blue) and poor (red) prognosis groups. **(b)** Boxplot of median centered gene expression value in the 64 discovery cohort cases, red (poor outcome) and blue (good outcome) groups determined by VF clustering. Wilcoxon rank-sum test *p*-value: * - $p < 0.05$, ** - $p < 0.01$.

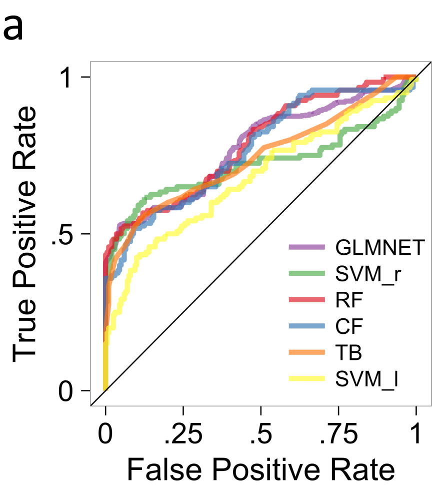

b

	Discovery Cohort				
	Accuracy	Kappa	Sensitivity	Specificity	AUC
**GLMNET**	0.794	0.522	0.955	0.525	0.78
**SVM_r**	0.781	0.498	0.93	0.533	0.72
**RF**	0.772	0.478	0.92	0.525	0.79
**CF**	0.747	0.388	0.975	0.367	0.77
**TB**	0.738	0.425	0.83	0.583	0.74
**SVM_l**	0.712	0.316	0.925	0.358	0.68

Fig. S14. ***Comparison of 6 prediction models fit with the 14GC to separate good and poor prognosis groups – as determined by the 9 VFs - in the discovery cohort.*** **(a)** Receiver operating characteristic curves for the performance of each classifier in the discovery cohort. **(b)** Summary of performance metrics of the 6 classifiers. Classifier abbreviations are: GLMNET - Generalized Linear Model with Elastic Net Regularization, SVM_r - radial kernel SVM, SVM_l - linear kernel SVM, TB - Tree Bagger, RF - Random Forest, CF - C-Forest.

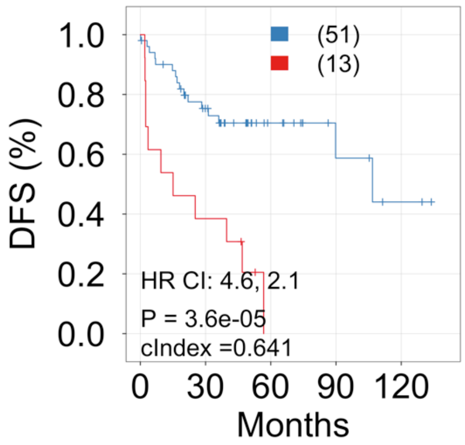

Fig. S15. ***Kaplan-Meier plot of 14GC good and poor prognosis groups in the discovery cohort.*** A GLMNET classifier was trained with the 64 discovery cohort cases, and then applied to the same discovery cohort cases to separate them into good and poor survival groups. The survival times for these two groups was visualized in a Kaplan-Meier plot.

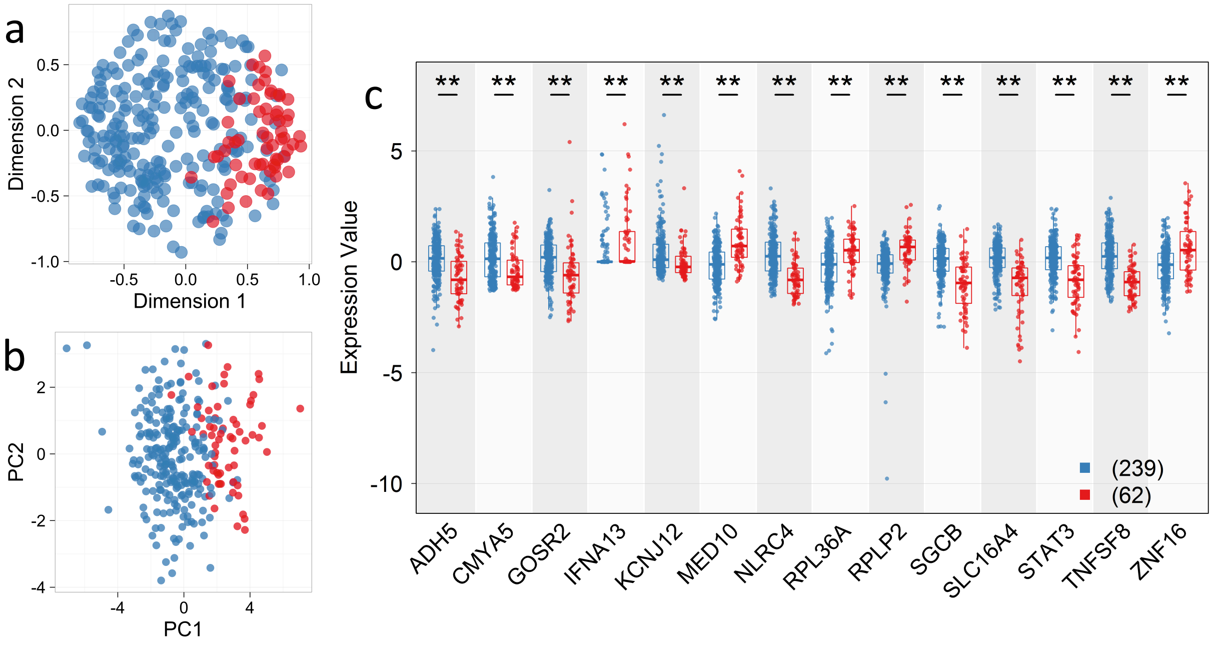

Fig. S16. ***Visualization of expression levels of the 14GC in the validation cohort (n = 301).*** **(a)** Multidimensional scaling plot (average correlation distance) of cases colored by the GLMNET classification with the 14-gene panel (14GC). **(b)** The first two principle components of 14 genes in the validation cohort. Colors correspond to good (blue) and poor (red) prognosis group classified by 14GC. The first two principle components explain 39.8% of the variance. **(c)** Boxplot of median centered gene expression value in the good (blue) and poor (red) outcomes groups determined by 14GC. Wilcoxon rank-sum test p-value: * - $p < 0.05$, ** - $p < 0.01$

Supplementary Tables

Table S1. List of nuclear morphology and texture features from CRImage (excel spreadsheet)

Table S2: Image Analysis Performance Metrics

**Computation time – 1 Tile (3,000px^2)**		
Nuclear Segmentation	5 min	
Nuclear Classification	<1 min	
Vasculature Area Classification	2 min	
**Nuclear classification in image tiles from archival cases (HMMSeg annotated)**		
	**Training**	**Validation**
# cases	8	8
# tumor nuclei	16,660 (70.8%)	170,825 (67%)
# endothelial nuclei	6,224 (26.4%)	75,058 (29.4%)
# inflammatory nuclei	669 (2.8%)	9,317 (3.6%)
Total	23,553	255,200
**Nuclear classification performance**		
Tumor cells sensitivity/specificity	0.933 / 0.87	
Endothelial cells sensitivity/specificity	0.87 / 0.9	
**Vascular area classifier performance**		
Area used for training (CD31 positive + negative)		6,000 pixels
CD31 positive validation area		>5 x 10^8 pixels
Overlap Index	$\frac{\lvert CD31 \cup VAM \rvert}{\min(\lvert CD31 \rvert, \lvert VAM \rvert)}$	0.64 ± 0.08
Accuracy	$\frac{Raw\ agreement}{\#\ Testing\ points}$	0.74 ± 0.03
Area under ROC curve		0.79

Table S2. ***Training and validation set metrics for nuclear and vascular area classifiers.*** Analysis was performed on a dual Intel Xeon (E5-2630 v2 @ 2.60GHz) workstation with 32GB RAM and running 64-bit Windows 7 Enterprise. GPU accelerated tasks, implemented by MATLAB's gpuArray data type, were performed on an NVIDIA Quadro K4000. In application, the processing time for one tile was approximately 8 minutes after time consuming intermediates were pre-processed. Cellular feature extraction and eosin mask preprocessing added considerably to the processing time per tile.

Table S3: Characteristics of patient cohorts				
	**Local Development**	**TCGA Discovery**	**TCGA Validation**	***P* (Chi-square)**
**Number of cases**				
	8	64	301	-
**Fuhrman Grade**				
**1**	0	3 (4.7%)	4 (1.3%)	0.192
**2**	5	32 (50%)	129 (43%)	
**3**	3	23 (35.9%)	121 (40.3%)	
**4**	0	6 (9.4%)	42 (14%)	
**N/A**	0	NA	4 (1.3%)	
**Median Age (min – max)**				
	n/a	60 (33 - 86)	59 (29 - 86)	-
**AJCCC Stage**				
**Stage I**	n/a	33 (51.6%)	153 (50.8%)	0.244
**Stage II**	n/a	12 (18.8%)	37 (12.3%)	
**Stage III**	n/a	18 (28.1%)	110 (36.5%)	
**Stage IV**	n/a	1 (1.6%)	1 (0.3%)	
**N/A**	n/a	NA	NA	
**Number recurred**				
	n/a	27 (42.2%)	87 (28.9%)	0.185
**Median Time to Recurrence (months)**				
	n/a	89.8	123.7	-
**Tiles analyzed (@ 750 um^2 / tile)**				
	204	2,714	0	-

Table S3. ***Clinical variables for patient cohorts.*** 380 cases had complete recurrence status and RNA-seq data. Of these, 12 had received neoadjuvant therapy. Three of the remaining were normal samples. The remainder were divided into the 64 case discovery and 301 case validation cohorts. Median time to recurrence was defined as the time point at which the right-censored Kaplan-Meier plot intersected the line survival rate = 0.5. Chi-square test confirmed there is no significant difference in the frequency of grade, stage, or recurrence of cases between discovery and validation set.

Table S4. 182 unique genes correlated with vascular features. (excel spreadsheet)

Table S5	Gene name	Activity	Disease association	REF
CMYA5	Cardiomyopathy-associated 5	Desmin binding Vesicular transport	Cardiomyopathy schizophrenia	17, 18
STAT3	Signal transducer and activator of transcription 3	Signal transduction Gene transcription	Angiogenesis Vascular leakage	19, 20
ADH5	alcohol dehydrogenase 5	Opposes NO signaling, protein denitrosylation	Impaired cardiovascular function	21
NLRC4	NLR family CARD domain containing 4	Innate immunity inflammosome	Inflammatory disease, infantile enterocolitis	22, 23
RPL36A	Ribosomal protein L36	60S ribosomal subunit Translational regulation	Hepatocellular carcinoma	24
RPLP2	ribosomal protein lateral stalk subunit P2	Phosphoprotein involved in protein elongation	Upregulated in many cancers	25, 26
SLC16A4	Solute carrier family 16 member 4	Monocarboxylate transporter for pH and energy homeostasis	Prognostic biomarker in ccRCC	27
TNFSF8	tumor necrosis factor superfamily member 8	CD30 ligand	Inflammation	28
ZNF16	zinc finger protein 16	Transcription factor	Erythroid and megakaryocyte differentiation	29
IFNA13	interferon alpha 13	Inflammatory/reproductive cytokine	Downregulated in dilated cardiomyopathy	30
SGCB	Sarcoglycan beta	Dystrophin complex, sarcoglycan transport	Limb-girdle muscular dystrophy cardiomyopathy	31
KCNJ12	potassium voltage-gated channel subfamily J member 12	Repolarization of cardiac muscle	Dilated cardiomyopathy	32
MED10	mediator complex subunit 10	RNA Pol-II transcriptional regulation	Heart valve development	33
GOSR2	golgi SNAP receptor complex member 2	Vesicular trafficking	Familial essential hypertension	34, 35
**Prognostic renal cancer biomarker**		**Association with vascular or heart biology**		**Cancer association**

Table S5. Functional annotation of genes in 14 gene classifier (14GC). Annotation was performed manually through PubMed.

Name	Grade	Stage	Gene34	14VF	14GT	ScoreTest	RatioTest	AIC	C-Index
Grade	1.71					1.16	1.17	478.96	0.58
Grade + CC34	1.38		2.66			3.21	3.12	469.96	0.67
Grade + 14VF	1.75			3.53		5.22	4.27	464.68	0.65
Grade + 14GT	1.32				4.26	5.84	3.68	467.39	0.64
Stage		4.00				6.62	5.83	459.14	0.70
Stage + CC34		3.34	2.18			7.35	6.54	454.22	0.75
Stage + 14VF		3.97		3.47		10.10	8.44	445.46	0.74
Stage + 14GT		3.51			3.65	10.28	7.51	449.72	0.74
Grade + Stage	1.19	3.81				5.86	5.10	460.82	0.70
Grade + Stage + CC34	0.96	3.38	2.20			6.68	5.89	456.20	0.76
Grade + Stage + 14VF	1.16	3.79		3.46		9.45	7.78	447.23	0.73
Grade + Stage + 14GT	1.00	3.50			3.65	9.54	6.83	451.72	0.73

Table S6. ***Multivariate Cox regression models for disease free survival.*** Performance of uni- and multivariate Cox regression models in a 207 case validation cohort of TCGA cases. Grade and stage were converted to bivariate predictors (1,2 vs 3,4 for both). CC34 is group designation by Clear Code 34 gene expression. 14VF is group designation by a GLMNET gene classifier trained on VF-risk groups. 14GT is group designation by a GLMNET gene classifier trained on 24-months disease free status. The model variables are listed in the first column. Grade, Stage, CC34, 14VF and 14GT indicate the individual hazard ratios from each multivariate model (blue bars). Each of the multivariate models was also assessed for goodness of fit by the Score Test, C-Index, Ratio Test, and Akaike's Information Criterion (AIC).

Table S7: Description of 9 VFs

**1. EC Density (Kurtosis)**	Endothelial cell density was measured as the area of endothelial nuclei divided by the tile area, in pixels. The kurtosis describes how outlier-prone the distribution is. Expression of this feature was increased in good relative to poor outcome cases.						
Gene	CMYA5	STAT3					
Correlation	0.405	0.410					
**2. Arm Orientation SD (Stdv)**	Orientation of the arms were measured in each tile relative to a horizontal line. For each tile the standard deviation was calculated. A distribution of binned standard deviations was analyzed for each case. Standard deviations of these case-level distributions were higher for poor than good outcomes cases. This suggests greater disorganization of vascular arms in poor outcomes cases.						
Gene	ADH5	NLRC4	RPL36A	RPLP2	SLC16A4	TNFSF8	ZNF16
Correlation	-0.424	-0.427	0.426	0.467	-0.455	-0.428	0.463
**3. Arm Number (Kurtosis)**	Kurtosis of the distribution of the number of arms from each tile. Case level distributions had higher kurtosis in the good prognosis relative to poor prognosis cases. This suggests less variability in the number of arms per tile in the good prognosis cases.						
**4. Arm Number (Skewness)**	Skewness of the distribution of the number of arms from each tile. Case level distributions had higher skewness in the good prognosis relative to poor prognosis cases. This suggests asymmetry in the distribution, where a positive value indicates a greater frequency of tiles with larger numbers of arms.						
Gene	IFNA13						
Correlation	-0.328						
**5. BP Lacunarity (Stdv)**	Standard deviation of the lacunarity of branching points. Higher lacunarity, or more “gappiness” in the branching point organization, was associated with poor outcomes.						
Gene	SGCB						
Correlation	-0.402						
**6. EC Lacunarity (Stdv)**	Standard deviation of the lacunarity of endothelial cells. Higher lacunarity, or more “gappiness” in the endothelial cell organization, was associated with poor outcomes.						
**7. Arm Lacunarity (Kurtosis)**	Kurtosis of the lacunarity of vascular arms. Higher lacunarity, or more “gappiness” in the arm organization, was associated with the good outcome group. This suggests that some cases in the good prognosis group have large areas without vasculature.						
Gene	KCNJ12	MED10					
Correlation	0.417	-0.318					
**8. EC Density (Stdv)**	Endothelial cell density was measured as the area of endothelial cell nuclei divided by the tile area, in pixels. Greater variability in endothelial cell density was observed in the poor outcome relative to good outcome group.						
Gene	GOSR2						
Correlation	-0.465						
**9. EC Density (Skewness)**	Endothelial cell density was measured as the area of endothelial cell nuclei divided by the tile area, in pixels. Skewness of the endothelial cell density to the right of the normal distribution was greater in the good prognosis relative to poor prognosis group.						

Table S7. ***Nine VFs used in case stratification in the discovery cohort.*** To the right of each VF are conceptual summaries. The entries underneath each bold-faced VF title indicate the correlated genes that were selected by information gain, and their respective correlation coefficients (Spearman's rho). An entry of 'None' indicates that no genes correlated with that gene were selected for final gene signature classification.

Supplementary Data Files:

Data file S1. Demonstration code for Hidden Markov Model segmentation in IHC images. Included is an example data set, example images, and instructions to install and run the scripts. The demonstration implementation is in R.

Data file S2. Demonstration code for Vascular Area Mask segmentation from H&E images. Included is an example data set and a minimal working script with the method self-contained. The demonstration implementation is in Matlab.

Data file S3. Vascular features and disease free survival groups of cases in the TCGA discovery and validation cohorts.

Supplementary References:

1. Cerami E, Gao J, Dogrusoz U, Gross BE, Sumer SO, Aksoy BA, et al. The cBio Cancer Genomics Portal: An Open Platform for Exploring Multidimensional Cancer Genomics Data. Cancer Discov. 2012; 2(5): 401-404.
2. Gao J, Aksoy BA, Dogrusoz U, Dresdner G, Gross B, Sumer SO, et al. Integrative Analysis of Complex Cancer Genomics and Clinical Profiles Using the cBioPortal. Sci Signal. 2013 6(269): pl1.
3. Saeys Y, Inza I, Larranaga P. A review of feature selection techniques in bioinformatics. Bioinformatics. 2007; 23: 2507-2517.
4. O. Taramasco, S. Bauer, RHMM: Hidden Markov Model simulations and estimations, R package, 2.0.3 (2013).
5. D. Meyer, F.T. Wein, Support vector machines. The interface to libsvm in package e1071, (2015).
6. G. Pau, F. Fuchs, O. Skylar, M. Boutros, W. Huber, EBImage – an R package for image processing with applications to cellular phenotypes. *Bioinformatics* **26**, 979-981 (2010).
7. P. Perona, J. Malik, Scale-space and edge detection using anisotropic diffusion. *IEEE Transactions on pattern analysis and machine intelligence* **12**, 629-639 (1990).
8. R Team, R: A language environment for statistical computing. (2013).
9. P. Romanski, FSeclector: Selecting attributes. *R package version 0.18*, (2009).
10. A. Peters, T. Hothorn, Package 'ipred' *R package version 0.8-7*, (2009).
11. A. Liaw, M. Wiener, Classification and regression by randomForest. *R news* **2**, 18-22 (2002).

12. C. Strobl, A.L. Boulesteix, T. Kneib, T. Augustin, A. Zeileis, Conditional variable importance for random forests. *BMC Bioinformatics* **9**, 307 (2008).

13. C. Strobl, A.L. Boulesteix, A. Zeileis, T. Hothorn, Bias in random forest variable importance measures: illustrations, sources and a solution. *BMC Bioinformatics* **8**, 25 (2007).

14. T. Hothorn, P. Buhlmann, S. Dudoit, A. Molinaro, M.J. van der Laan, Survival ensembles. *Biostatistics* **7**, 355-373 (2006).

15. J. Friedman, T. Hastie, R. Tibshirani, glmnet: lasso and elastic net regularized generalized linear models. *R package* (2013).

16. X. Robin, N. Turck, A. Hainard, N. Tiberti, F. Lisacek, J.C. Sanchez, M. Muller, pROC: an open-source package for R and S+ to analyze and compare ROC curves. *BMC Bioinformatics* 12, 1 (2011).

17. Sarparanta J. Biology of myospryn: what's known? J Muscle Res Cell Motil. 2008; 29(6-8): 177-180.

18. Chen X, Lee G, Maher BS, Fanous AH, Chen J, Zhao Z, et al. GWA study data mining and independent replication identify cardiomyopathy-associated 5 (CMYA5) as a risk gene for schizophrenia. Mol Psychiatry. 2011; 16(11): 1117-1129.

19. Dutzmann J, Daniel JM, Bauersachs J, Hilfiker-Kleiner D, Sedding DG. Emerging translational approaches to target STAT3 signaling and its impact on vascular disease. Cardiovasc Res. 2015; 106(3): 365-374.

20. Yun JH, Park SW, Kim KJ, Bae JS, Lee EH, Paek SH, et al. Endothelial STAT3 Activation Increases Vascular Leakage Through Downregulating Tight Junction Proteins: Implications for Diabetic Retinopathy. J Cell Physiol. 2017; 232(5): 1123-1134.

21. Beigi F, Gonzalez DR, Minhas KM, Sun QA, Foster MW, Khan SA, et al. Dynamic denitrosylation via S-nitrosoglutathione reductase regulates cardiovascular function. Proc Natl Acad Sci USA. 2012; 109(11): 4314-4319.

22. von Moltke J, Ayres JS, Kofoed EM, Chavarria-Smith J, Vance RE. Recognition of bacteria by inflammasomes. Annu Rev Immunol. 2013; 31: 73-106.

23. Romberg N, Al Moussawi K, Nelson-Williams C, Stiegler AL, Loring E, Choi M, et al. Mutation of NLRC4 causes a syndrome of enterocolitis and autoinflammation. Nat Genet. 2014; 46(10): 1135-1139.

24. Song MJ, Jung CK, Park CH, Hur W, Choi JE, Bae SH, et al. RPL36 as a prognostic marker in hepatocellular carcinoma. Pathol Int. 2011; 61(11): 638-644.

25. Gardner-Thorpe J, Ito H, Ashley SW, Whang EE. Ribosomal protein P2: a potential molecular target for antisense therapy of human malignancies. Anticancer Res. 2003; 23(6C): 4549-4560.

26. Artero-Castro A, Castellvi J, Garcia A, Hernandez J, Ramon y Cajal S, Lleonart ME. Expression of the ribosomal proteins Rplp0, Rplp1, and Rplp2 in gynecologic tumors. Hum Pathol. 2011; 42(2): 194-203.

27. Fisel P, Stuhler V, Bedke J, Winter S, Rausch S, Hennenlotter J, et al. MCT4 surpasses the prognostic relevance of the ancillary protein CD147 in clear cell renal cell carcinoma. Oncotarget. 2015; 6(31): 30615-27.

28. Kennedy MK, Willis CR, Armitage RJ. Deciphering CD30 ligand biology and its role in humoral immunity. Immunology. 2006; 118(2): 143-152.

29. Deng MJ, Li XB, Peng H, Zhang JW. Identification of the trans-activation domain and the nuclear location signals of human zinc finger protein HZF1 (ZNF16). Mol Biotechnol. 2010; 44(2): 83-89.

30. Golovleva I, Biasotto M, Verpy E, Roos G, Meo T, Tosi M, et al. Novel variants of human IFN-alpha detected in tumoral cell lines and biopsy specimens. J Interferon Cytokine Res. 1997; 17(10): 637-645.

31. Barresi R, Di Blasi C, Negri T, Brugnoni R, Vitali A, Felisari G, et al. Disruption of heart sarcoglycan complex and severe cardiomyopathy caused by beta sarcoglycan mutations. J Med Genet. 2000; 37(2): 102-107.

32. Szuts V, Menesi D, Varga-Orvos Z, Zvara A, Houshmand N, Bitay M, et al. Altered expression of genes for Kir ion channels in dilated cardiomyopathy. Can J Physiol Pharmacol. 2013; 91(8): 648-656.

33. Just S, Hirth S, Berger IM, Fishman MC, Rottbauer W. The mediator complex subunit Med10 regulates heart valve formation in zebrafish by controlling Tbx2b-mediated Has2 expression and cardiac jelly formation. Biochem Biophys Res Commun. 2016; 477(4): 581-588.

34. Meyer TE, Shiffman D, Morrison AC, Rowland CM, Louie JZ, Bare LA, et al. GOSR2 Lys67Arg is associated with hypertension in whites. Am J Hypertens. 2009; 22(2): 163-168.

35. Pan S, Nakayama T, Sato N, Izumi Y, Soma M, Aoi N, et al. A haplotype of the GOSR2 gene is associated with essential hypertension in Japanese men. Clin Biochem. 2013; 46(9): 760-765.
