# Supplementary material for: A novel machine learning approach reveals latent vascular phenotypes predictive of renal cancer outcome
Source: Sci Rep. 2017 Oct 16;7:13190. doi: 10.1038/s41598-017-13196-4 (PMC5643431; doi:10.1038/s41598-017-13196-4)
Supplement: Supplementary file 4 — Supplementary Data 1 [file 41598_2017_13196_MOESM4_ESM.zip › Supplementary_Data_S1.zip!/HMMseg_0.2/HMMseg.pdf]

HMMSeg v.0.1

Andrew Conley[1] and Nathan Ing[2]

[1,2]Cedars-Sinai Medical Center, Los Angeles, CA, USA

September 2, 2016

1 Introduction

HMMSeg is a set of R programs intended for segmentation of chromogens stained in Immunohistochemistry slides for subsequent analysis or re-application in a steralized label-matrix format. It was created for use by those with familiarity in Digital Image Processing and Analysis and the R programming environment. Generally, it operates on the R-G-B channels found in standard JPEG-compression family file types. There is a focus on what is called whole slide processing, specifically on files generated by an Aperio scanner in `SVS` file format. However, it remains completely possible to apply our procedures to standalone `TIFF` and `JPEG` images.

This package is a parser for translating stained slides into label matrices, wherein a single value corresponds to a class of IHC stain chromogens. Since we work primarily with RGB values, it is crucial that the prepared slide's chromogens are readily separable in RGB space, otherwise the results of `HMMSeg` may be compromised. For example, slides stained with Red and Brown may experience false positive Red class assignments in places of light Brown staining. That said, it is possible to work with this data if appropriate precautions are implemented.

In this document we'll review the source code and the core steps for going from an IHC stained slide to an annotated label matrix, and finally for transferring these annotations to H&E data as in our publication `paper_URL_here.com`. A formal package version with some routines for use in standardized data is underway.

2 Dependencies

First obtain the package files here: `http:\permananet_link_goes_here.com` or clone the Git repository: `http:\link_to_my_github.com`. I'm running `R version 3.3.1 (2016-06-21)`.

For working with SVS files we use Bio-Formats command line tools (Open Microscopy Environment) which may be downloaded from here[1 2]. Make note of where you unzip the files. SVS format function also uses the package stringr. The classifiers are built around the packages e1071, and RHmm[3]. RHmm 2.0.3 was downloaded from a legacy repository[4] and installed:

```
install.packages('/path/to/RHmm_2.0.3.tar.gz', repo=NULL, type='source',
    dependencies=TRUE)}
```

Some image processing routines provided in the packages CRImage and EBImage, available through Bioconductor[5]. We operate on many individual tiles which represents a so-called embarassingly parallel task. Parallelization is implemented in the package doParallel. That's it. Launch R and source in the essential contents. All this code is in example_script.R.

```
setwd("~/HMMseg/code")
source("constructInitialData.R")
source("makeImageClassifierHMM.R")
source("removeSmallRegions.R")
source("makeOutlines.R")
source("classifyImageStainingHMM.R")
source("classifySVS.R")

> ls()
[1] "classifyImageStainingHMM" "classifySVS"
[3] "constructInitialData"  "makeImageClassifierHMM"
[5] "makeOutlines"          "removeSmallRegions"
```

3 Classify an IHC image: Quick version

We'll work through the example data provided in the data folder. There are two CSV's in /HMMSeg/data/csv/: observations.csv and labels.csv which define the color classes and values we'll use to train the Hidden Markov Model and SVM classifiers. As a point of reference our example data has 15,000 data points corresponding to 6 classes: white, light blue, dark blue, brown, light brown and purple. The function constructInitialData provides a go-between for getting these CSV's into the format we'll use going forward. To do a new training and see how it works, do the section — section name —- instead.

[1] http://www.openmicroscopy.org/site/support/bio-formats5.2/users/comlinetools/

[2] Please also have an updated version of Java as the default

[3] On an OSX 10.11.5 system a compilation error was experienced, possibly associated with Rcpp and the RHmm package. The solution was to first install an update as described: http://thecoatlessprofessor.com/programming/rcpp-rcpparmadillo-and-os-x-mavericks-lgfortran-and-lquadmath-error/

[4] https://cran.r-project.org/src/contrib/Archive/RHmm/

[5] http://bioconductor.org

```
setwd("~/HMMseg/data/csv")
trainingData <- constructInitialData("./observations.csv",
    "./labels.csv")

class(trainingData)
[1] "list"
> length(trainingData)
[1] 6
```

You'll also find a series of TIF images in the folder `/HMMSeg/data/images`. These are small crops from 40X slides we'll use to train and test the classification. We'll use these first then move on to that bigger `SVS` image also in the `/images` directory. We'll provide the parameters `trainingData` and `initialImage` to the function `makeImageClassifierHMM`.

```
setwd("~/HMMseg/data/images")

> dir()
 [1] "01.tif" "02.tif" "03.tif" "04.tif" "05.tif" "06.tif" "07.tif"
 [8] "08.tif" "09.tif" "10.tif"

library(EBImage)
img = readImage("./01.tif")

display(img)

classifiers <- makeImageClassifierHMM(trainingData=trainingData,
                                      initialImage=img,
                                      verbose=TRUE)

> length(classifiers)
[1] 2
> names(classifiers)
[1] "HMM" "SVM"
```

You'll see that `classifiers` is a list of two objects: an `RHmm` model, and an `e1071` multi-class SVM. The reason for this duality is that the bulk of classification is done by RHmm's Viterbi algorithm, supported by an SVM for improved accuracy. We classify, in turn rows of the image, then columns of the same image. Then, pixels with disagreements between the two results are resolved with our SVM classifier. The workhorse function, `classifyImageStainingHMM` outputs both these row and column images so this may be made clearer in example.

```
dir.create('./results')
img = readImage("./01.tif")
```

```
stateImg <- classifyImageStainingHMM(image = img,
    hmm = classifiers$HMM,
    classifier = classifiers$SVM,
    cleanUp = TRUE,
    excessStates = c(1),
    saveProcess = TRUE,
    saveDirectory = './results',
    verbose = TRUE)
```

In our new `results` directory, there should be three images: row image, column image and cleaned up agreement and outline images (suffixed with `r`, `c`, `a` and `o`, respectively). We requested `cleanUp = TRUE`, so simple morphological transformations were applied to remove small speckles, fill tiny holes, and perform a degree of smoothing to the output. The first state was defined to be the optical background, so it's it during clean up. In practice this can be accomplished at any stage after, or you may even want to retain the background areas. In pactice, the function `removeSmallRegions` may be replaced with whatever post-processing procedures befit your needs. Similarly, `makeOutlines` is for visualization purposes only.

Let's apply our classifier to multiple TIF's with a simple loop. The only change is the addition of the `savePrefix` option to appropriately name each of our output files according to their original names.

```
imgs = dir(path = '.', pattern = '*.tif')
for(k in 1:length(imgs)) {
    im_name = imgs[[k]]
    im = readImage(im_name, convert = TRUE)
    stateImg <- classifyImageStainingHMM(image = im,
        hmm = classifiers$HMM,
        classifier = classifiers$SVM,
        cleanUp = TRUE,
        excessStates = c(1),
        saveProcess = TRUE,
        saveDirectory = './results',
        savePrefix = sub('.tif', '', im_name),
        verbose = TRUE)
}
```

Now let's apply our same classifier to a larger `SVS` format slide image. Included is not a whole slide, but a small section 6,000 x 6,000 pixels at 40X which is about 1,500μm^2. Let's set the `windowSize = 300`. You'll need the directory where you've unpacked the `bftools` binaries, and where all the `HMMseg` code lives.

```
file = '/path/to/HMMseg/data/images/001.svs'
classifySVS(file = file, hmm = classifiers$HMM,
           classifier = classifiers$SVM,
```

```
windowSize = 500,
saveProcess = TRUE,
savePrefix = '001',
saveDirectory = '/path/to/HMMseg/data/images/results',
threads = 4,
svstoolsloc = '/path/to/bftools',
HMMsegloc = '/path/to/HMMseg/code',
verbose=TRUE)
```
